# Vitamin D_3_ and deconvoluting a rash

**DOI:** 10.1172/jci.insight.163789

**Published:** 2023-01-24

**Authors:** Madison K. Ernst, Spencer T. Evans, Jose-Marc Techner, Robert M. Rothbaum, Luisa F. Christensen, Ummiye Venus Onay, Dauren Biyashev, Michael M. Demczuk, Cuong V. Nguyen, Kord S. Honda, Thomas S. McCormick, Lam C. Tsoi, Johann E. Gudjonsson, Kevin D. Cooper, Kurt Q. Lu

**Affiliations:** 1 Department of Dermatology, Northwestern University Feinberg School of Medicine, Chicago, Illinois, USA.; 2 Department of Dermatology, University Hospitals Cleveland Medical Center, Case Western Reserve University & Veterans Affairs Medical Center, Cleveland, Ohio, USA.; 3 Department of dermatology, University of Michigan, Ann Arbor, Michigan, USA.

**Keywords:** Clinical Trials, Dermatology, Bioinformatics, Cellular immune response, Skin

## Abstract

**BACKGROUND:**

Adverse drug reactions are unpredictable immunologic events presenting frequent challenges to clinical management. Systemically administered cholecalciferol (vitamin D_3_) has immunomodulatory properties. In this randomized, double-blinded, placebo-controlled interventional trial of healthy human adults, we investigated the clinical and molecular immunomodulatory effects of a single high dose of oral vitamin D_3_ on an experimentally induced chemical rash.

**METHODS:**

Skin inflammation was induced with topical nitrogen mustard (NM) in 28 participants. Participant-specific inflammatory responses to NM alone were characterized using clinical measures, serum studies, and skin tissue analysis over the next week. All participants underwent repeat NM exposure to the opposite arm and then received placebo or 200,000 IU cholecalciferol intervention. The complete rash reaction was followed by multi-omic analysis, clinical measures, and serum studies over 6 weeks.

**RESULTS:**

Cholecalciferol mitigated acute inflammation in all participants and achieved 6 weeks of durable responses. Integrative analysis of skin and blood identified an unexpected divergence in response severity to NM, corroborated by systemic neutrophilia and significant histopathologic and clinical differences. Multi-omic and pathway analyses revealed a 3-biomarker signature (CCL20, CCL2, CXCL8) unique to exaggerated responders that is suppressed by cholecalciferol and implicates IL-17 signaling involvement.

**CONCLUSION:**

High-dose systemic cholecalciferol may be an effective treatment for severe reactions to topical chemotherapy. Our findings have broad implications for cholecalciferol as an antiinflammatory intervention against the development of exaggerated immune responses.

**TRIAL REGISTRATION:**

clinicaltrials.gov (NCT02968446).

**FUNDING:**

NIH and National Institute of Arthritis and Musculoskeletal and Skin Diseases (NIAMS; grants U01AR064144, U01AR071168, P30 AR075049, U54 AR079795, and P30 AR039750 (CWRU)).

## Introduction

Rashes, drug reactions, and allergic skin responses are encountered in everyday medical practice. The skin is a complex organ, maintained by tight homeostatic control of native and infiltrating cells. Local skin injury can trigger a reactionary cascade leading to a multisystem response. Cutaneous adverse drug eruptions, especially to topical chemotherapeutics, present a frequent iatrogenic challenge to both patients and clinician: 30%–80% of patients develop reactions severe enough to warrant treatment discontinuation (1–3). There are currently no reliable molecular markers to predict these reactions and no effective therapies beyond medication discontinuation, which may interfere with treatment of the underlying disease (4).

Cholecalciferol (vitamin D_3_ [VitD_3_]) has recently emerged as a critical immunomodulatory hormone with regulatory roles in the innate and adaptive immune systems (5). The biologically active form of VitD, calcitriol (1,25[OH]_2_D_3_), binds to the ubiquitously expressed VitD receptor and has mechanisms of inflammatory control including expansion of antiinflammatory M2 macrophages, inhibition of proinflammatory NF-κB signaling, and upregulation of arginase-1, an antiinflammatory factor involved in tissue repair (6–8). Pharmacologic use of VitD has traditionally been directed toward long-term dietary supplementation. However, VitD is increasingly used as a low-risk therapeutic agent. Topical VitD_3_ analogs (i.e., calcipotriene) are an effective adjuvant treatment of chronic psoriasis (9). While orally administered VitD allows for more direct systemic effects due to higher, more precise dosing, it may also have clinical utility as a low-risk therapeutic agent in the acute setting. Additionally, high-dose oral VitD treatment has been found to accelerate the resolution of sunburn-induced inflammation in humans (10) and mitigates the severity of initial chemical injury and subsequent inflammation in mice without adverse effects (8, 11).

In the present study, we aimed to investigate whether a single intervention of high-dose oral VitD_3_ affected an experimentally induced rash at both the clinical and molecular levels in healthy human adults. The rash-inducing agent was nitrogen mustard (NM), a topical irritant and FDA-approved chemotherapeutic agent with well-known potential to cause systemic inflammatory reactions and contact dermatitis in high doses (12). We first applied NM to all participants without VitD intervention to establish a comprehensive clinical and molecular inflammatory profile for the NM reaction alone. We then repeated the NM application to each participant’s contralateral arm and randomized participants to receive either a 1-time, high dose of oral VitD or placebo (Supplemental Figure 1A; supplemental material available online with this article; https://doi.org/10.1172/jci.insight.163789DS1). The complete rash reaction was followed via clinical measures, serum studies, and skin tissue analysis collected in 14 visits over 8 weeks, providing matched, multipoint comparisons. These data demonstrate that a 1-time, high oral dose of VitD administered immediately after the cutaneous application of NM reduces inflammation by clinical, histologic, and multi-omic parameters at acute (3-day) and longitudinal (6-week) follow up.

During our analysis, we also discovered a striking divergence in the degree of participants’ inflammatory responses to low-dose topical NM. While it is well established that topical chemotherapeutics may induce treatment-limiting reactions secondary to hypersensitivity and immunologic intolerance in some patients undergoing longitudinal therapy, these reactions are often attributed to the concentration, total dose, or formulation of the medication and typically develop over time with repeated applications (13, 14). Unfortunately, there is no pretest or reliable way currently to predict which patients will go on to develop these reactions. Here we report an observable divergence in the immunologic response to topical chemotherapy that occurs at both an earlier time and with lower doses than previously believed. Pathway enrichment analysis conducted from RNA-Seq and proteomic data suggests that the severe response is associated with IL-17 signaling, which has not previously been implicated in the development of hypersensitivity reactions. We subsequently identified a 3-protein signature that was consistently upregulated in participants who would go on to have a severe response that was not apparent in participants who would continue to have a mild response. We suggest that this signature may have predictive clinical utility in the real-world setting. Such a tool would help identify at-risk patients and allow clinicians to make early treatment adjustments or implement prophylactic supportive therapies.

## Results

### Participant disposition and baseline characteristics.

**Figure 1:**
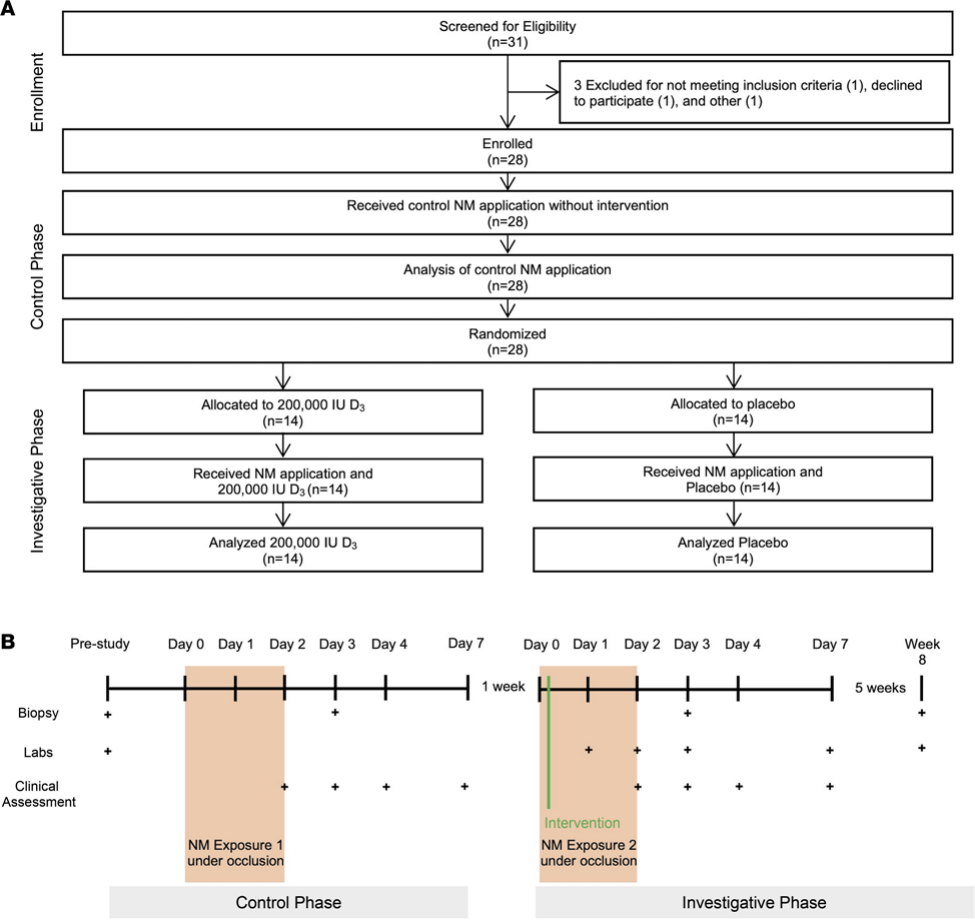
Study design. (**A**) The study progression, including enrollment, allocation, and analysis of participants. (**B**) A schematic of the biphasic, parallel study design. Before study initiation, an 8 mm punch biopsy was performed (Bx_0) and serum labs were drawn for serum D_3_ metabolites, serum phosphorous level, complete blood count with differential, and a comprehensive metabolic panel. All participants were then exposed to nitrogen mustard (NM) under occlusion for 48 hours without subsequent intervention. Clinical assessment, including skin erythema by chromometer and thickness measurements by bifold caliper, were conducted on days 2, 3, 4, and 7. A punch biopsy was obtained on day 3 (Bx_1). Participants returned 1 week after the conclusion of the control phase to begin the investigative phase. Participants were randomized 1:1 to receive either 200,000 IU D_3_ or placebo 3 hours after NM application to the previously untreated arm. Serum labs were drawn on days 1, 2, 3, and 7. Clinical assessments were performed on days 2, 3, 4, and 7. Biopsy was obtained on day 3 (Bx_2). Participants returned for follow-up 6 weeks after investigative phase initiation, during which a final biopsy (Bx_3) and labs were taken.

This 8-week, randomized, double-blinded, placebo-controlled interventional trial included 28 healthy participants and occurred on a rolling basis between September 2017 and August 2018 (Figure 1). The longitudinal trial design allowed participants to act as their own controls. The 2 treatment groups had similar baseline characteristics, except for a female predominance in the VitD(–) group (Supplemental Table 1).

### Safety and tolerability.

The dose of NM applied to each participant (approximately 0.033 g/application) is 200,000 times less than the total amount applied during an average mycosis fungoides cutaneous T cell lymphoma (MF-CTCL) treatment and was not expected to induce significant adverse reaction. PRO-Diary wristwatch results identified minor adverse effects of NM application, including mild itch, burning sensation, pain, irritation, and warmth at the application site. No adverse effects warranted participant withdrawal or exclusion from the study; however, 1 participant from both the VitD(+) and the VitD(–) groups developed bullae in the investigative phase.

VitD intervention was well tolerated with no adverse effects. Serum VitD-metabolite levels remained under the level of potential toxicity. For VitD(+) participants, the average serum 25(OH)D_3_ was 41 ng/mL (SEM = 2.4) at 24 hours and 52 ng/mL (SEM = 3.0) at 7 days. The postulated level of toxicity in adults is 150 ng/mL (15). The highest 25(OH)D_3_ value measured was 77.1 ng/mL. Serum levels of the active VitD metabolite 1,25(OH)_2_D_3_ were also significantly increased in VitD(+) participants compared with baseline (x̄ = 46.5 pg/mL, SEM = 1.5) and VitD(–) participants at all matched time points (*P* < 0.001 for all; 24-hour VitD x̄ = 79.8 pg/mL, SEM = 4.8; 7-day x̄ = 72.2 pg/mL, SEM = 3.6). Serum 1,25(OH)_2_D_3_ levels peaked 24 hours after VitD administration. VitD(–) participant 1,25(OH)_2_D_3_ levels did not differ from baseline (x̄ _placebo_ = 51.3, SEM = 3.2; *P* = 0.15) (Supplemental Table 2). Calcium and phosphorus levels remained within reference ranges.

### Oral VitD reduces inflammatory marker expression both acutely and longitudinally.

**Figure 2:**
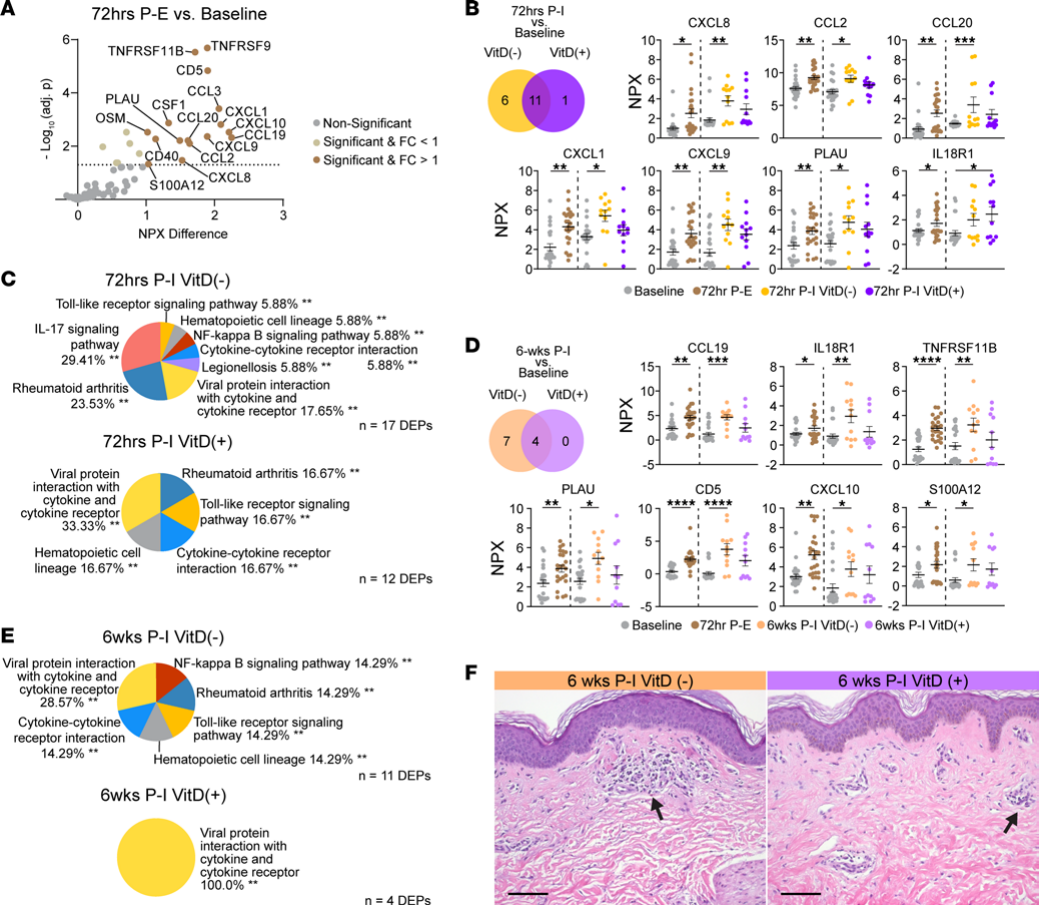
Vitamin D mitigates acute inflammation with durable effects and suppresses markers of NM injury involved in IL-17 signaling pathways. (**A**) Volcano plot of mean normalized protein expression (NPX) changes for differentially expressed proteins (DEPs) between samples taken 72hr P-E (*n* = 24) and baseline (*n* = 24) determined by paired *t* tests. (**B**) Venn diagram shows the number of DEPs found in **A** that are upregulated 72hrs P-I in VitD(–) (*n* = 12) and VitD(+) (*n* = 12) relative to baseline (*n* = 24). Scatter plots show mean ± SEM of NPX values for labeled groups. (**C**) Pie charts show the percent composition of significantly enriched KEGG pathways using DEP lists from **B** as input from comparisons between 72hrs P-I VitD(+) and VitD(–) relative to baseline. (**D**) Venn diagram shows the number of DEPs found in **A** that are upregulated 6wks P-I in VitD(–) (*n* = 12) and VitD(+) (*n* = 11) relative to baseline. Scatter plots show mean ± SEM of NPX values for labeled groups. (**E**) Pie charts show the percent composition of significantly enriched KEGG pathways using total DEP lists as input from comparisons between 6wks P-I VitD(+) and VitD(–) relative to baseline. (**F**) Representative H&E staining from biopsies taken 6wks P-I. Scale bar: 100 μm. Arrows denote areas of inflammation in the dermis. (**B** and **D**) Significance was determined by linear mixed-effects modeling followed by Benjamini-Hochberg correction for multiple comparisons. *P_adj_ < 0.05,**P_adj_ < 0.01,***P_adj_ < 0.001,****P_adj_ < 0.0001. (**C** and **E**): Significant enrichment was determined by a 2-sided hypergeometric test and p-values were corrected using Bonferroni step down. **pV < 0.01.

Before investigating the effect of VitD on cutaneous inflammatory injury, we established the multi-omic expression profile resulting from NM injury without intervention at 72 hours after exposure (72hrs P-E). Proximity extension assays that quantify a curated panel of 92 inflammatory proteins identified 22 significantly (*P*_adj_ < 0.05) differentially expressed proteins (DEPs) at 72hrs P-E compared with baseline, of which 16 had an NPX difference > 1 (Figure 2A). These 22 DEPs represent the inflammatory protein profile resulting from NM injury (NM injury markers). They include tumor necrosis factor receptors (TNFRSF9, TNFRSF11B/OPG), chemokines (CCL2/MCP1, CCL3, CCL19, CCL20, CXCL1, CXCL8/IL8, CXCL9, CXCL10), ILs (OSM, IL-6, IL-7), and others (CD40, CD5, CSF1, uPA/PLAU, EN-RAGE/S100A12, DNER, SCF, IL-18R1, CX3CL1). Pathway enrichment for these markers in the KEGG database revealed enrichment for the IL-17 signaling pathway (Supplemental Figure 2A). Additionally, there were 405 significant differentially expressed genes (DEGs; log_2_ fold change [log_2_FC] cutoff = 1, *P*_adj_ < 0.005) at 72hrs P-E compared with baseline (Supplemental Table 3). KEGG pathway enrichment for the 323 upregulated DEGs revealed IL-17 signaling, TLR signaling, and NF-κB signaling (Supplemental Figure 2B).

We then sought to characterize the acute effects of high-dose VitD intervention by analyzing DEGs and DEPs at 72 hours after intervention (72hrs P-I) relative to baseline. VitD(–) participants had 331 upregulated and 57 downregulated DEGs (*S* < 0.005, log_2_FC cutoff = 2) at 72hrs P-I. At the same time point, VitD(+) participants had 427 upregulated and 80 downregulated DEGs relative to baseline (Supplemental Table 3). Proteomic analysis from the same time point revealed that VitD(–) participants upregulated 17 of the 22 NM injury associated DEPs. By comparison, VitD(+) participants upregulated 12 NM injury markers, only 1 of which was not also upregulated among VitD(–) participants (Figure 2B). Pathway enrichment analysis of the NM injury markers upregulated in VitD(–) participants demonstrated enrichment of TLR signaling, NF-κB signaling, and IL-17 signaling (Figure 2C). Of the 6 NM injury markers uniquely upregulated by 72hrs P-I VitD(–) participants, 4 markers — CCL20, CXCL1, CCL2, and CXCL8 — contributed to IL-17 signaling pathway enrichment. NM injury markers upregulated in VitD(+) participants did not demonstrate enrichment of IL-17 signaling pathways. These results suggest that acute NM exposure–induced IL-17 signaling may be suppressed by orally administered VitD.

To characterize the longitudinal effects of VitD intervention on NM-induced skin injury, we assessed DEGs (Supplemental Table 3) and DEPs in samples taken 6 weeks after NM injury and VitD intervention (6 weeks [6wks] P-I). After 6 weeks, VitD(+) participants upregulated 67% fewer NM injury markers than they did 72hrs P-I VitD(+). In comparison, VitD(–) participants only saw a 35% reduction at 6wks compared with 72hrs P-I (Figure 2D). The 7 NM injury markers expressed in VitD(–) but not VitD(+) participants at 6wks P-I were CCL19, CXCL9, CD5, TNFRSF11B, CXCL10, S100A12, and PLAU. Pathway analysis of 6wks P-I VitD(–) NM injury markers reveals persistent enrichment of TLR signaling and NF-κB signaling that is resolved in VitD(+) (Figure 2E and Supplemental Figure 2C). Interestingly, the inflammatory molecular differences between intervention groups did not translate to clinically significant differences in skin redness, edema, or histology at any time point (Figure 2F).

### Identification of an intervention-independent exaggerated immune response to chemical exposure.

While adverse reactions to NM are common in high doses, the overall risk for severe skin reactions was hypothetically minimized in this study by using a low total dose of a topical NM gel that was FDA approved specifically for its lower incidence of adverse skin reactions (13). Thus, we did not have a priori expectations that participant responses to NM would differ significantly within our study population.

**Figure 3:**
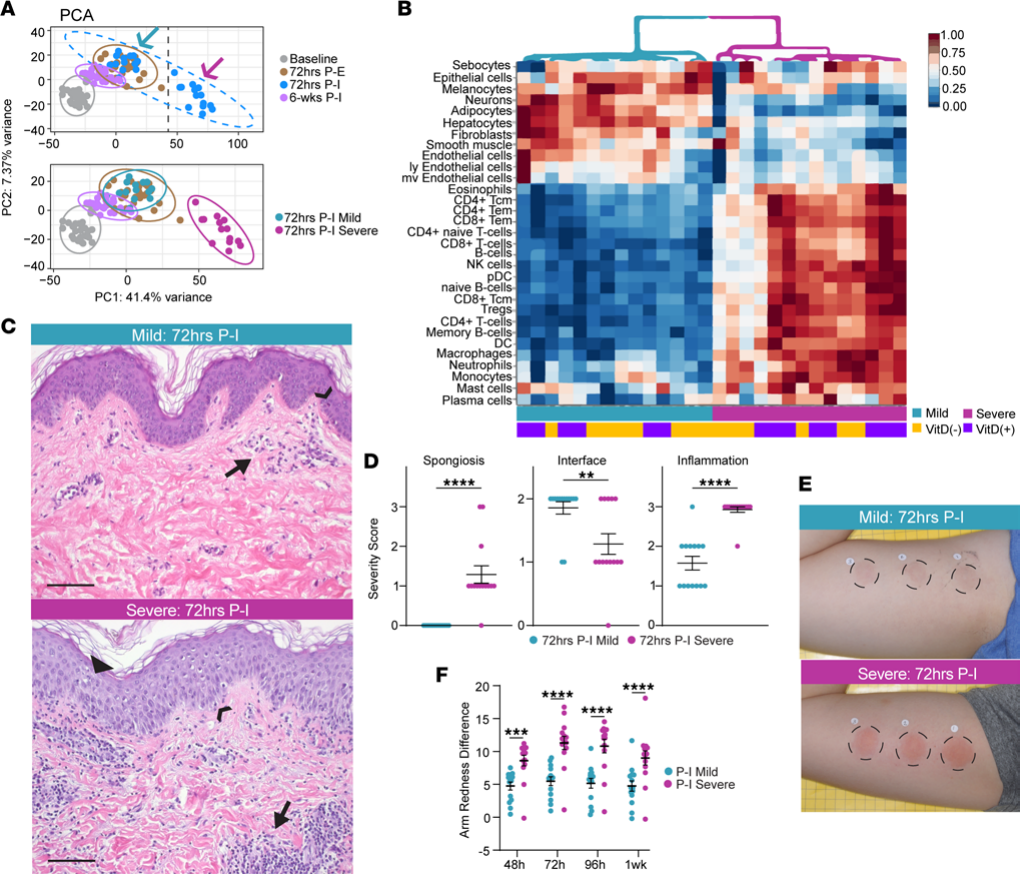
Repeat NM exposure reveals an intervention-independent divergent inflammatory response. (**A**) Principal component analysis (PCA) of RNA-Seq data from all samples (*n* = 110) colored by time of biopsy collection with corresponding 95% CI ellipses shown. Arrows denote 2 distinct clusters within the 72hrs P-I samples (*n* = 28) whose 95% CI ellipses are shown in the subsequent PCA. (**B**) Heatmap of percent transformed cell type enrichment scores for 72hrs P-I RNA-Seq counts. Column dendrogram denotes unsupervised clustering of samples, and corresponding group membership of samples is shown below. (**C**) Representative H&E staining of 72hrs P-I Mild (*n* = 14) and Severe (*n* = 14) responders. Examples of spongiosis, degree of interface change, and inflammation are denoted by the triangle, arrowhead, and arrow, respectively. Scale bar: 100 μm. (**D**) Dot plots of severity scores for degree of spongiosis, interface change, and inflammation observed on H&E sections taken 72hrs P-I. Data are shown as mean ± SEM. Unpaired *t* tests were used to determine statistical significance. **P < 0.01, ****P < 0.0001. (**E**) Representative gross images of Mild and Severe participants’ arms 72hrs P-I. Dashed circles denote NM exposure site. (**F**) Dot plot of arm redness differences between exposed and unexposed sites taken after second NM exposure. Two-way ANOVA followed by Tukey’s multiple-comparison test were used to determine statistical significance ***P_adj_ < 0.001, ****P_adj_ < 0.0001. mv, mitral valve; ly, lymphocytic; Tcm, central memory T cell; Tem, effector memory T cell; pDC, plasmacytoid DCs.

When we performed principal component analysis (PCA) with the RNA-Seq data, we found that samples taken from the same time point after NM exposure clustered together, as expected (Figure 3A). However, within the cluster of samples taken 72hrs P-I, 2 distinct subgroups were observed. This observed variance was not explained by intervention group or demographic differences (Supplemental Figure 3A). Notably, 1 of these subgroups clustered with 72hrs P-E samples (Figure 3A, blue arrow), while another clustered separately along the PC1 axis in the positive direction (PC1^+^) (Figure 3A, pink arrow), indicating the presence of an intervention-independent factor driving RNA-Seq variability. Given that PC1^+^ was associated with immune and inflammatory pathway enrichment according to PCA2GO analysis (Supplemental Table 4), we suspected that the subgroup clustering further along this axis had experienced an exaggerated inflammatory response to NM. Thus, we labeled this group “Severe,” and we labeled the subgroup that clustered with 72hrs P-E samples “Mild.”

Differential gene expression analysis at 72hrs P-I revealed that Severe participants upregulated 1,105 genes and downregulated 964 more genes (*P*_adj_ < 0.05, log2FC cutoff = 1) than Mild participants. Grouping these ‘Severe’ DEGs according to their *Z* score–transformed expression profile across biopsies identified gene clusters involved in the response to NM-induced injury (Supplemental Figure 3B). Corresponding KEGG pathway enrichment of these DEG clusters found significant enrichment for inflammatory pathways, including Th17 cell differentiation, Th1 and Th2 cell differentiation, T cell receptor signaling, NK cell–mediated cytotoxicity, NF-κB signaling, and IL-17 signaling (Supplemental Figure 3C).

To characterize the tissue milieu in 72hrs P-I samples, we used xCell (16), a cell type enrichment analysis webtool, to deconvolute bulk RNA-Seq data and identify enriched cell types. Unsupervised clustering of samples by cell type enrichment results revealed a striking separation into 2 groups (Figure 3B) that demonstrated a 100% agreement with the Severe and Mild subgroups identified by PCA in Figure 3A. Immune cells, including B cells, CD4^+^ and CD8^+^ T cells, NK cells, neutrophils, and macrophages, were more enriched among Severe samples. Native skin cells, including sebocytes, epithelial cells, endothelial cells, fibroblasts, and melanocytes, were more enriched among Mild samples. These results validate the suspected divergence in inflammatory response between Mild and Severe at 72hrs P-I.

To understand how Mild and Severe responses manifested clinically, we first evaluated histologic sections taken from both response groups. We observed that Severe responders tended to have more immune cell infiltration and less dermal-epidermal junction barrier integrity (Figure 3C). Observed differences were quantified and confirmed using blinded histologic analysis by a board-certified dermatopathologist, who scored the degree of inflammation, spongiosis (a routine dermatopathology measure of intracellular edema within the epidermis), and interface change for each specimen using a standardized 0–3 severity scale. Specimens from 72hrs P-I Severe participants received significantly higher severity scores for inflammation (x̄ _severe_ = 2.93, SEM = 0.07; x̄_mild_= 1.57, SEM = 0.17; *P* < 0.0001) and spongiosis (x̄ _severe_ = 1 .29, SEM = 0.22; x̄ _mild_= 0, SEM = 0; *P* < 0.0001) compared with Mild (Figure 3D). Phenotypically, Severe participants demonstrated increased redness at the site of NM exposure (Figure 3E). Quantified redness difference between NM-exposed and nonexposed skin was significantly higher in P-I Severe compared with P-I Mild participants at all measured time points (Figure 3F). Severe participants also had significantly higher skin thickness — a proxy for skin edema — by bifold caliper measurements (x̄ _severe_ = 2.8mm; x̄ _mild_ = 1.9 mm; *P* = 0.05). The agreement between the PCA and DEG pathway enrichment analysis, cell type enrichment analysis, histologic scoring, and clinical assessments provides clear evidence for an intervention-independent divergence in response severity to NM between the P-I Severe and P-I Mild subgroups.

### Predictive markers of the exaggerated response to NM enrich IL-17 signaling.

**Figure 4:**
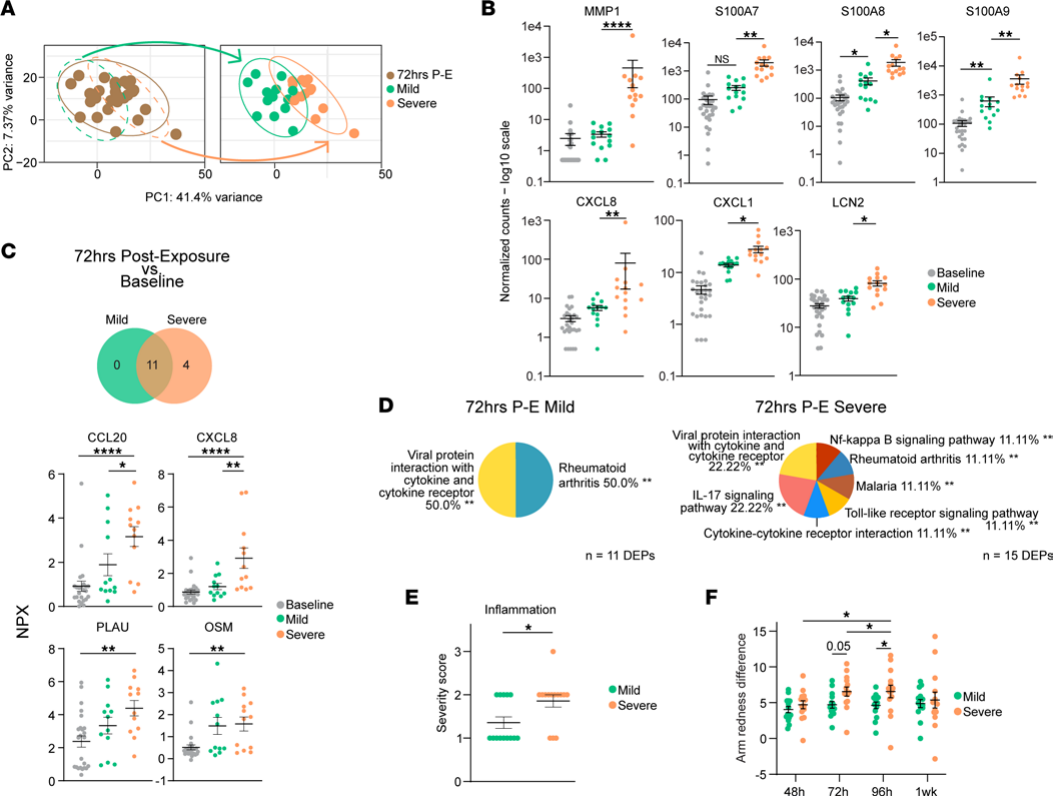
IL-17 signaling during initial NM exposure is involved in predisposing participants to an exaggerated response upon subsequent NM exposure. (**A**) PCA of samples taken 72hrs P-E (*n* = 28). Green and orange arrows show where Mild (*n* = 14) and Severe (*n* = 14) participants fall within the total group. The 95% CI ellipses for 72hrs P-E Mild and Severe are shown. (**B**) Dot plots of DEGs increased (log_2_FC > 1) in 72hrs P-E Severe relative to Mild participants that enrich the IL-17 signaling pathway. Significance was determined by Wald test followed by Benjamini-Hochberg correction for multiple comparisons; *P_adj_ < 0.05, **P_adj_ < 0.01, ***P_adj_ < 0.001, ****P_adj_ < 0.0001. (**C**) Venn diagram of NM injury DEPs (NPX > 1) in Mild (*n* = 12) and Severe (*n* = 12) groups at 72hrs P-E relative to baseline (*n* = 24). Dot plots show mean NPX; data are shown as mean ± SEM, with error bars of DEPs uniquely increased in 72hrs P-E Severe relative to baseline. Significance was determined by linear mixed-effects modeling followed by Benjamini-Hochberg correction for multiple comparisons; *P_adj_ < 0.05, **P_adj_ < 0.01, ****P_adj_ < 0.0001. (**D**) Pie charts show the percent composition of significantly enriched KEGG pathways using NM injury DEP lists as input from comparisons between 72hrs P-E Severe and Mild relative to baseline. Significant enrichment was determined by a 2-sided hypergeometric test, and *P* values were corrected using Bonferroni step down. **pV < 0.01. (**E**) Dot plots of severity score (scores 0–3) means; data are shown as mean ± SEM, with error bars in H&E sections taken 72hrs P-I and grouped by response severity. Unpaired *t* tests were used to determine statistical significance; *P < 0.05. (**F**) Dot plot of arm redness differences between exposed and unexposed sites taken after first NM exposure. Two-way ANOVA followed by Tukey’s multiple-comparison test were used to determine statistical significance; *P_adj_ < 0.05.

Mild and Severe NM response types were initially identified from data obtained during the intervention phase of this study (participants’ second NM exposure). However, because this divergence was determined to be intervention independent, we hypothesized that a participant’s fated response type was determined prior to the second NM exposure. Thus, we conducted a “look-back” analysis to determine if Mild and Severe responders could be identified in the control phase of this study (participants’ first NM exposure). Identification of early markers for a severe response fate may have utility in predicting the clinical response to NM. By labeling 72hrs P-E samples in the PCA according to their 72hrs P-I Mild or Severe designations (Figure 4A), we observed the same orientation of 95% confidence ellipses and pattern of clustering as seen in Figure 3A, though to a lesser extent. As with 72hrs P-I samples, 72hrs P-E Severe samples clustered further along the PC1 axis than 72hrs P-E Mild.

Differential expression analysis with RNA-Seq data demonstrated 42 significantly upregulated and 23 downregulated (log_2_FC cutoff = 1; *P*_adj_ < 0.05) genes in Severe participants relative to Mild at 72hrs P-E (Supplemental Table 3). KEGG pathway enrichment analysis with the upregulated genes revealed IL-17 signaling enrichment (Supplemental Table 5). The DEGs responsible for IL-17 signaling enrichment were *MMP1*, *CXCL8*, *CXCL1*, *S100A8*, *S100A7*, *S100A9*, and *LCN2* (Figure 4B). Protein quantification analysis comparing 72hrs P-E to baseline for Mild and Severe subgroups revealed 11 and 15 DEPs, respectively (Figure 4C). Four DEPs — CCL20, CXCL8, PLAU, and OSM — were uniquely upregulated among 72hrs P-E relative to baseline (Figure 4C); therefore, they may serve as predictive biomarkers for developing a severe response to iterative NM exposures. KEGG pathway enrichment analysis of the 15 NM injury markers upregulated among 72hrs P-E Severe revealed IL-17 signaling (Figure 4D) due to 5 markers: CCL2, CCL20, CXCL1, CXCL8, and CXCL10.

In agreement with the DEG and DEP findings, 72hrs P-E Severe demonstrated higher clinical measures of inflammation than 72hrs P-E Mild. Higher severity scores for inflammation (Figure 4E, x¯ _severe_ = 1.85; x̄ _mild_ = 1.36; *P* = 0.016) and increased redness between NM-exposed and unexposed skin at 72hrs P-E (*P* < 0.05), trending through 96hrs P-E (Figure 4F, *P* = 0.052), were found in Severe.

These results suggest that IL-17 signaling may be involved in priming the body to mount a severe response to subsequent NM exposures. The 72hrs P-E Severe participants expressed early inflammatory biomarkers involved in IL-17 signaling, such as CXCL8 and CCL20, which may have utility as a diagnostic panel for predicting adverse clinical reactions.

### VitD suppresses inflammation in the context of both mild and exaggerated inflammatory reactions.

We initially established in Figure 2 that, for all participants after injury, fewer inflammatory markers were expressed among VitD(+) than VitD(–) participants. Given the divergence of response characterized in Figure 3 and validated in Figure 4, we sought to determine how VitD intervention affects each response-severity group. Though our study was not powered for subgroup analysis, we observed that VitD intervention provided an antiinflammatory benefit in both the Mild and Severe contexts.

**Figure 5:**
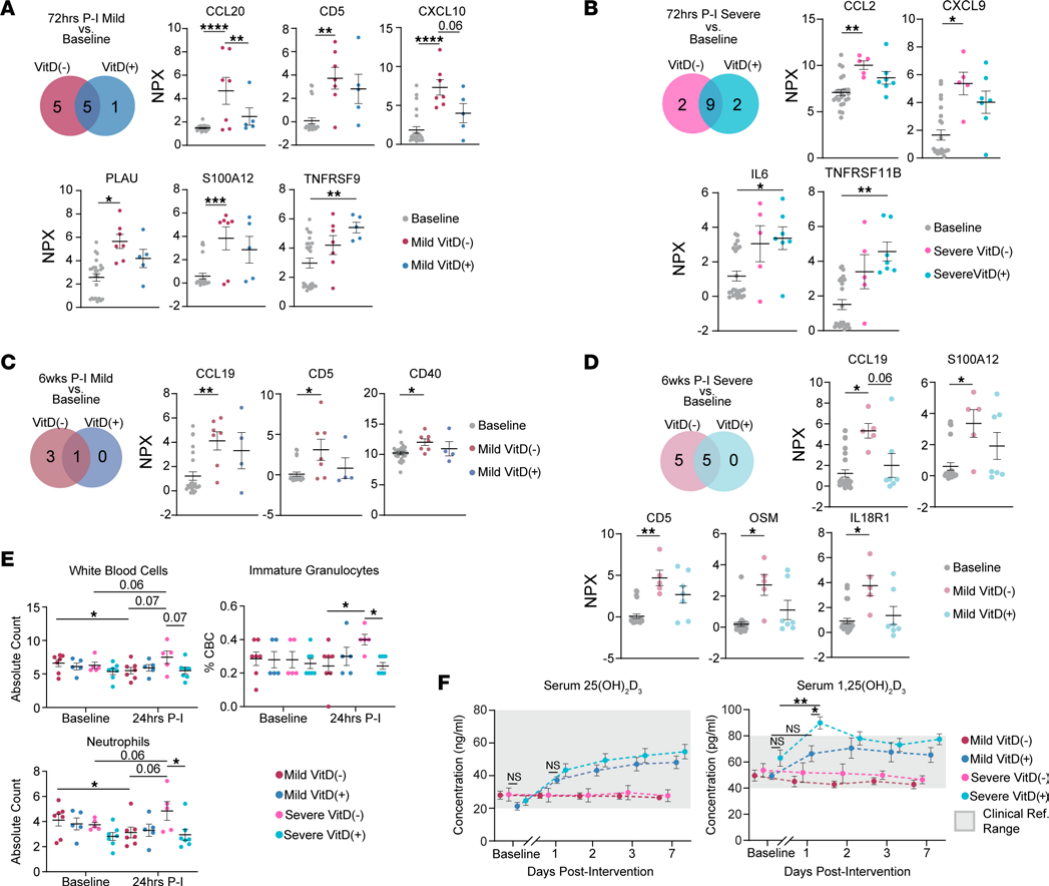
Vitamin D reduces inflammation, despite an exaggerated response to NM exposure. (**A**) Venn diagram of the number of NM injury DEPs that are upregulated 72hrs P-I in Mild VitD(–) (*n* = 7) and VitD(+) (*n* = 5) relative to baseline (*n* = 24). Data are shown as mean ± SEM of NPX values for labeled groups. (**B**) Venn diagram shows the number of NM injury DEPs that are upregulated 72hrs P-I in Severe VitD(–) (*n* = 5) and VitD(+) (*n* = 7) relative to baseline. Data are shown as mean ± SEM of NPX values for labeled groups. (**C**) Venn diagram shows the number of NM injury DEPs that are upregulated 6wks P-I in Mild VitD(–) (*n* = 4) and VitD(+) (*n* = 7) relative to baseline. Data are shown as mean ± SEM of NPX values for labeled groups. (**D**) Venn diagram shows the number of NM injury DEPs that are upregulated 6wks P-I in Severe VitD(–) (*n* = 7) and VitD(+) (*n* = 5) relative to baseline. Data are shown as mean ± SEM of NPX values for labeled groups. (**E**) Dot plots of mean CBC levels; data are shown as mean ± SEM, with error bars at baseline and 24-hours P-I in Mild VitD(–) (*n* = 7), Mild VitD(+) (*n* = 5), Severe VitD(–) (*n* = 5), and Severe VitD(+) (*n* = 7). Two-way ANOVA followed by Tukey’s multiple-comparison test were used to determine statistical significance. *P < 0.05. (**F**) Dot plots over time of vitamin D metabolites 25(OH)_2_D_3_ and 1,25(OH)_2_D_3_. Two-way ANOVA followed by Tukey’s multiple-comparison test were used to determine statistical significance. *P_adj_ < 0.05, **P_adj_ < 0.01. (**A**–**D**) Significance was determined by linear mixed-effects modeling followed by Benjamini-Hochberg correction for multiple comparisons; *P_adj_ < 0.05, **P_adj_ < 0.01, ***P_adj_ < 0.001, ****P_adj_ < 0.0001.

Among Mild participants at 72hrs P-I, those who were VitD(–) upregulated 10 NM injury markers relative to baseline. Comparatively, Mild VitD(+) participants upregulated only 6 NM injury markers (Figure 5A). Of the 5 NM injury markers upregulated only in VitD(–) participants, CCL20 and CXCL10 enriched for IL-17 signaling (Supplemental Figure 4). TNFRSF9, an inducible mediator of CD4^+^ and CD8^+^ T cell survival and function (17–20), was the only NM injury marker expressed in VitD(+) but not VitD(–) at 72hrs P-I (Figure 5A).

At 72hrs P-I, Severe VitD(–) and VitD(+) participants each expressed 11 NM injury markers, 9 of which were the same markers (Figure 5B). The 2 NM injury markers upregulated only among VitD(–) were CCL2, a monocyte recruitment factor (21), and CXCL9, a T cell chemokine secreted by activated macrophages and keratinocytes (22, 23). Pathway enrichment analyses of the upregulated DEPs from 72hrs P-I Severe VitD(–) and VitD(+) were similar due to the significant overlap in pathway membership between CCL2 and IL-6 (Supplemental Figure 4, C and D, and Supplemental Table 5). Upregulation of promonocyte factors is a well-established response to high-dose NM in animal models (24–26).

Among 6wks P-I Mild participants, 3 NM injury markers (CCL19, CD5, and CD40) remained persistently upregulated in VitD(–) participants but not VitD(+) participants (Figure 5C), suggesting a greater degree of NM injury resolution in VitD(+) participants. In 6wks P-I Severe, 5 NM injury markers (CCL19, CD5, S100A12, OSM, and IL18R1) remained persistently upregulated only in VitD(–) participants (Figure 5D). Taken together, these results suggest that VitD intervention mitigates inflammatory protein upregulation and reduces NM injury markers among both Mild and Severe participants in the acute phase and longitudinal follow-up.

The upregulation of inflammatory cytokines would conceivably lead to changes in immunologic parameters during real-world, high-dose exposure to NM. However, we did not anticipate that the low experimental dose of NM used in this study would induce an inflammatory response strong enough to create measurable systemic change. Nevertheless, Severe VitD(–) participants demonstrated a trend toward neutrophilia after the second NM exposure, evidenced by an increase (*P* = 0.06) in absolute neutrophil count (ANC) 24hrs P-I relative to baseline (Figure 5E). Additionally, Severe VitD(–) participants had a trend toward increased absolute leukocyte counts (*P* = 0.06) relative to baseline. Relative to Severe VitD(+) participants at 24hrs P-I, Severe VitD(–) participants had increased ANC (*P* = 0.03) and percent immature granulocytes (*P* = 0.04). The differential complete blood count (CBC), comprehensive metabolic panel (CMP), and serum phosphorous levels for VitD(+) participants were within normal limits and unchanged from baseline.

Serum studies also suggest that the metabolism of exogenous, high-dose VitD may differ depending on the recipient’s underlying inflammatory profile. Under normal physiologic conditions, serum 1,25(OH)_2_D_3_ production and catabolism are tightly regulated (27). Notably, among VitD(+) participants, we found that Severe participants experienced a significant rise in serum 1,25(OH)_2_D_3_ above clinical reference range 24hrs P-I that was not observed in their Mild counterparts (Figure 5F).

## Discussion

**Figure 6:**
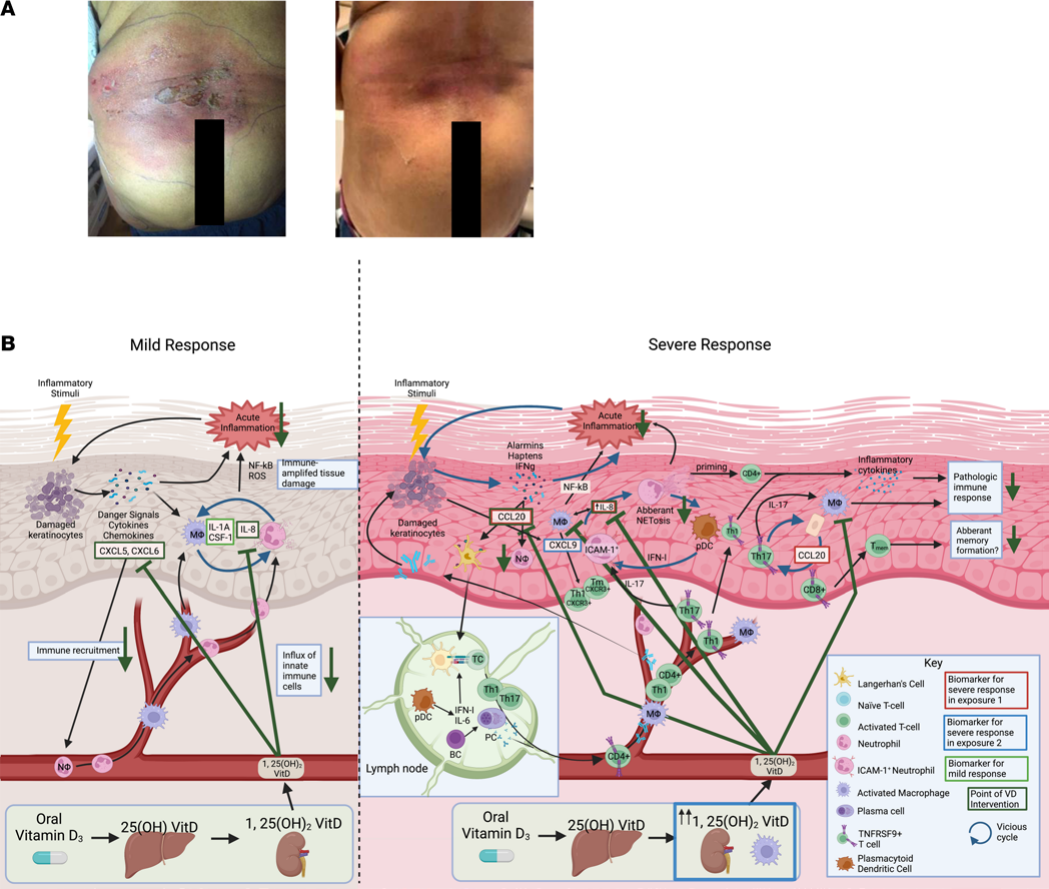
Clinical vignette and proposed mechanism of NM injury and VitD intervention. (**A**) Exaggerated inflammatory response to topical NM therapy in a patient with Stage IA CTCL before (left) and 5 days after (right) a single dose of 100,000 IU oral VitD and twice daily topical clobetasol 0.05% ointment. (**B**) Theoretical, simplified mechanisms of NM injury and VitD intervention in Mild (right) and Severe responders (left). Initial keratinocyte damage leads to the release of proinflammatory mediators that recruit and activate immune cells. Macrophage activation stimulates iNOS and NF-κB signaling and secretion of inflammatory factors, such as CXCL8 and CSF1 (63–65). VitD mediates acute inflammation by suppressing CXCL5, CXCL6, and CXCL8 expression (Supplemental Table 6). We identified CCL20, CCL2, and CXCL8 as exaggerated response markers. CCL20 binds CCR6 to stimulate IL-17 signaling and attract lymphocytes and myeloid DCs (37). These APCs travel to secondary lymphoid tissues and activate T cells. CXCL8 is a neutrophilic chemokine known to induce neutrophil extracellular trap (NET) formation at high concentrations (39, 66, 67). pDCs, which do not normally respond to self-antigens, may become activated by NETs due to the colocalization of self-DNA with pDC activating factors (68–70). Activated pDCs secrete type-1 IFN at the local injury site, contributing to acute inflammation and priming mature neutrophils to favor NET fate (71). Activated pDCs in secondary lymphoid organs selectively induce Th1 and Th17 differentiation and activate B cells (68, 70, 72). The resulting antibody production exacerbates tissue injury and may induce NETs, creating a vicious inflammatory cycle (66). NETs have also been shown to mediate activation of both CD4^+^ and memory T cells by direct contact, lowering their activation threshold to specific antigens and suboptimal stimuli (69). IL-17 signaling further activates neutrophils, macrophages, and keratinocytes and perpetuates vicious inflammatory cycles potentially responsible for increased response severity. VitD suppresses these vicious cycles by suppressing CXCL8, CCL20, and CCL2 upregulation.

In the control phase of this study, we analyzed the protein expression profile of untreated NM skin injury and identified 22 protein markers of untreated NM injury. In the intervention phase, we repeated the NM exposure, after which participants were randomized to receive placebo or a single 200,000 IU dose of oral VitD. Oral VitD was selected over topical for more precise dosing and direct systemic effects, and its use was justified based on a previous dosage study demonstrating its efficacy in reducing expression of proinflammatory mediators after sunburn (10). Half of the participants demonstrated an exaggerated inflammatory response to NM exposure, which is consistent with real-world clinical observations. Despite the divergence in participant inflammatory response to NM, all VitD(+) participants expressed fewer chemical injury–associated protein markers and demonstrated less inflammation than VitD(–) participants by clinical and histologic measures at both 3-day follow-up and after complete clinical resolution of injury. These results are consistent with anecdotal clinical observations that oral VitD mitigates severe cutaneous reactions to topical NM in the setting of stage IA CTCL (Figure 6A). No currently available clinical tools can predict who will go on to have an exaggerated, potentially treatment-limiting response to NM. However, analyses from this study suggest that a protein expression panel of CCL20, CCL2, and CXCL8 may have predictive clinical utility for capturing severe phenotypic outcomes. Our data also suggest that this utility is mediated by IL-17 pathway signaling.

Our study confirms that NM injury markers previously only identified in animal models and in vitro human keratinocyte studies (CCL2, CCL3, CXCL1, CXCL10, CCL19, OSM, PLAU, and CXCL8) are translatable to in vivo human work (24–26, 28–32). To our knowledge, the upregulation of OSM, CCL20, TNFRSF9, CD40, CD5, and CSF1 in response to NM injury was previously unestablished. Pathway analysis revealed an association of these NM injury markers with IL-17 signaling. IL-17 signaling has not been previously implicated in acute NM injury. Furthermore, our results demonstrating that 4 of the 5 NM injury markers suppressed by VitD are involved in IL-17 signaling suggest that VitD mitigates NM-mediated injury by suppressing IL-17 signaling. Since current IL-17–inhibiting drugs take weeks to become effective, (33) VitD may have unique utility for acute IL-17 signaling suppression. Six weeks after intervention, more NM injury markers remained upregulated among VitD(–) than VitD(+) participants, demonstrating that VitD provides lasting benefits that have utility in the chronic setting. These results suggest that high-dose oral VitD could serve as a low-risk, antiinflammatory treatment in the setting of acute chemical skin injury.

The intervention-independent divergence in severity of inflammatory response observed in this study is consistent with the variable response to topical chemotherapeutics observed in clinical practice, in which some patients tolerate repeat exposures while others experience treatment-limiting adverse effects (1–3). While high-risk patients were previously unpredictable, subgroup analysis of samples from the first NM exposure in this study identified that a 3-protein signature of CXCL8 and CCL20 — and, to a lesser extent, CCL2 — were upregulated in those who would go on to have an exaggerated response to the second NM exposure. While specific markers of an exaggerated response to NM skin injury have not been previously identified in humans, our results are consistent with past data implicating neutrophilic, monocytic, and T cell–mediated processes in exaggerated responses to topical chemotherapeutics (8, 25, 34). All 3 markers are associated with both acute and chronic inflammatory processes. CCL2 (macrophage chemoattractant protein 1) is a potent chemoattractant for monocytes as well as T cells and DCs (35, 36). Previous work in mice identified CCL2 upregulation upon high-dose topical SM exposure (24). Cutaneous CCL20 (macrophage inflammatory protein 3) upregulation is associated with acute keratinocyte injury and recruits myeloid DCs and Th17 cells to the injury site (37). IL-17 and CCL20 signaling may be perpetuated by a feedback loop between Th17 cells and keratinocytes (Figure 6B). CXCL8 is a potent neutrophilic chemokine that may act as a chemoattractant for neutrophils at low concentrations and may induce adaptive-inflammatory processes, such as neutrophil extracellular trap formation (NETosis), at high, receptor-saturating concentrations (38, 39).

Since topical chemotherapy regimens require repeated applications in the clinical setting, we propose that a panel identifying the combined upregulation of these 3 markers after a patient’s initial NM exposure may have clinical utility for predicting which patients may develop the exaggerated response phenotype with continued use. Early identification of at-risk patients would allow clinicians to make treatment adjustments or prophylactically provide stronger supportive therapies. Recent advances in noninvasive protein collection, such as tape stripping, increase the practicality of such a panel.

The 3-marker Severe response signature may provide insight into the mechanisms underlying the exaggerated-response phenotype. Previously, acute NM-associated inflammation was assumed to be mediated primarily by innate immunity. However, all 3 Severe response markers function in bridging innate and adaptive immunity, including IL-17 signaling, which has been identified in previous mouse lung studies as a driving pathway of chronic SM inflammation (40). Cell-type enrichment also found higher local populations of cells involved in adaptive immunity and IL-17 signaling (e.g., CD4^+^ and CD8^+^ T cells, mast cells, and macrophages; refs. 41–47) in Severe response participants. Since Mild-response participants lacked IL-17 signaling enrichment, these 3 markers may implicate adaptive immune processes in driving the initiation or maintenance of the exaggerated response to NM injury.

Regardless of whether an exaggerated response to topical chemotherapy occurs, our results suggest that systemic VitD is an effective immunomodulatory intervention. Protein, pathway-enrichment data, and serum VitD metabolite levels provide evidence that VitD metabolism and antiinflammatory properties differ depending on the underlying inflammatory status of the patient. During a mild inflammatory response, VitD is metabolized within reference-range levels and suppresses the primarily neutrophilic cytokines CXCL5, CXCL6, and CXCL8, which may suppress inflammatory feedback cycles (Figure 6B). Interestingly, we found that, in exaggerated responders who received VitD, the serum concentration of active VitD peaks above reference range 24 hours after administration. This spike in active VitD was not expected, but it may have clinical utility in serving as an early marker for patients who are fated to experience an exaggerated response to topical chemotherapeutics.

While this study was not designed to evaluate the mechanisms of VitD metabolism, the upregulation of promonocytic DEPs and CBC changes observed in Severe participants may suggest that the metabolic differences in VitD are mediated in part by an increase in activated macrophages, which are well known to conduct extrarenal hydroxylation of 25(OH)D_3_ to active VitD (48, 49). Differential protein expression analysis showed that VitD intervention suppresses the upregulation of all 3 exaggerated-response markers, CXCL8, CCL2, and CCL20. If these markers are indeed driving the exaggerated response phenotype, then VitD may have preventative properties against the development of the exaggerated response phenotype, in addition to its general antiinflammatory effects after chemical injury.

In conclusion, we provide proof-of-concept evidence that a single administration of high-dose oral VitD safely and durably suppresses clinical and biological markers of postinjury inflammation. We identified participants experiencing an exaggerated inflammatory response to chemical injury and used exploratory outcomes and post hoc analysis to associate this phenotype with IL-17 signaling and a potentially predictive biomarker signature. Additional studies investigating the mechanisms of the observed divergent response to injury, optimal dosing regimens, and adjuvant treatment potential for VitD therapies are essential next steps.

## Methods

### Study design.

The study was composed of a total of 14 visits over 8 weeks (Figure 1B). At study initiation, baseline measures were obtained. Skin erythema (redness) was quantified using a CR300 Chroma Meter (Minolta). Skin thickness, a proxy for acute edema, was obtained using a Mitutoyo 9 mm dial caliper. Blood was drawn and processed at a Clinical Laboratory Improvement Amendments–approved (CLIA-approved) hospital facility to determine baseline serum 25(OH)D_3_ levels (the major circulating form of VitD), CBC with differential, CMP, and phosphorous levels.

Chemical skin injury was induced using the vesicating agent, NM, as a model. Topical NM in the form of FDA-approved Valchlor (mechlorethamine) 0.016% gel was applied under Finn chamber occlusion to 3 adjacent 8 mm areas of each participant’s inner brachial arm. Occlusion chambers, which were used to standardize the topical NM exposures among patients, were removed after 48 hours. The dose of NM applied to each participant (approximately 0.033 g/application) is 200,000 times less than the total amount applied during an average MF-CTCL treatment. It was selected to induce a low-grade chemical injury without significant adverse reactions. Repeat measurements of skin redness and thickness were performed 48, 72, 96, and 168 hours after NM application. Photos of the exposed sites were taken at each visit. At the 72-hour visit, an 8 mm punch biopsy was performed from one of the mechlorethamine sites and processed for histologic evaluation.

Two weeks after the initial NM application, an identical procedure was used to apply NM gel under occlusion to the previously unexposed arm of each participant. After application, participants in the control group received 4 oral placebo tablets. Those in the intervention group received 200,000 IU of 25(OH)D_3_ as four 50,000 IU oral tablets. This dose was selected based on a previous pilot study and justified for safety based on several previous studies utilizing high-dose oral VitD (10, 50, 51). Skin thickness and redness were measured at the same time points as above, with the addition of a 6-week follow-up time point. A skin biopsy was again performed 72 hours after NM application. Participants also had repeat blood testing of CBC, CMP, 25(OH)D_3_, and serum phosphorous levels at 24, 48, 72, and 168 hours and at a 6-week follow up. Participants were given PRO-Diary wrist-worn devices, which prompted them to record participative skin irritation, pain, and itching twice daily during both phases of the study.

### Primary and secondary outcomes.

The primary outcome was a composite outcome composed of clinical and molecular measures of inflammation. The clinical components of the primary outcome were skin erythema by CR300 Chroma Meter, skin thickness by Mitutoyo 9 mm dial caliper, and blinded histopathologic assessment of skin tissue biopsies by a broad-certified dermatopathologist. The molecular components of the primary outcome were the significant changes in RNA and protein expression by bulk-RNA-Seq and protein quantification (Olink Inflammation panel proximity extension assay). Primary outcome measures were collected as specified above.

As secondary outcomes, we recorded serum levels of the 3 major VitD metabolites (25[OH]D_3_, 1,25[OH]_2_D_3_, and 24,25[OH]_2_D_3_), serum calcium, serum phosphorous, and CBC with differential. Molecular secondary outcome measures were collected as specified above. Clinical secondary outcomes were collected using PRO-Diary wrist-worn devices, which prompted participants to provide participative ratings of skin irritation, pain, and itching twice a day throughout the 8-week trial.

### Participant recruitment and randomization.

Healthy adult participants of any Fitzpatrick skin type were recruited from March 2017 to June 2018 by IRB-approved advertisements (approved by IRB of Case Western Reserve University and Case Comprehensive Cancer Center [CASE 3416]) posted throughout the hospital and university and by referrals from other dermatologists. Ineligibility criteria included individuals aged < 17 years; individuals who were pregnant, nursing, or anticipate becoming pregnant within 3 months; individuals taking ketoconazole, colestipol, cholestyramine, phenobarbital, phenytoin, mineral oil, warfarin, aspirin, > 400 mg/day ibuprofen, > 220 mg/day naproxen sodium, ≥ 4,000 IU/day or 20,000 IU/week of VitD supplements, or illicit drugs; individuals undergoing treatment with chemotherapy, antibiotics, biologics, or immunosuppressants; or individuals with a BMI > 40. Thirty-one adults were screened for eligibility, and 28 were consented to participation. Given the pilot nature of the study design and the high level of involvement required of study participants, the sample size was determined primarily by recruitment constraints.

Participants were block randomized into either the VitD group (VitD[+]) or placebo group (VitD[–]) using a balanced assignment (Figure 1A). All participants who completed both phases of the study and received the study drug were included in the per-protocol analysis. Significance of the canonical pathways represents the likelihood that genes in the differentially expressed gene set map to a particular process or pathway more than expected by random chance alone. The Bonferroni correction for multiple comparisons was not utilized, given the small overall number of planned comparisons in the primary outcome analyses as well as the exploratory nature of the post hoc analyses performed to guide further investigation.

### Blinding.

Participants and investigators performing assessments were blinded to intervention assignments. After the first 4 participants, there was an interim unblinding to confirm intervention safety. Blinding was reestablished for the remaining 24 participants until after the main analyses were completed. During the 8-week active study period, adverse effects were monitored by a designated unblinded investigator, our statistician, and clinical pharmacist.

### Tissue processing.

Snap-frozen skin-punch biopsies were weighed and submerged in the necessary volume of TRIzol according to the manufacturer’s protocol (Thermo Fisher Scientific). The tissue was then disrupted and homogenized in a Qiagen Tissue PowerLyzer at 3,500 rpm for 30 seconds. Disruption and homogenization was repeated after a 1-minute cool down. RNA was extracted from the aqueous phase, while the interphase and organic phase were preserved for DNA and protein extraction, respectively. Biopsy specimens from 4 of the 28 enrolled participants were processed in a previous batch, in which the interphase and organic phase were not preserved.

### RNA-Seq.

The quality of reads, in FASTQ format, was evaluated using FastQC. Reads were trimmed to remove Illumina adapters from the 3’ ends using cutadapt (52). Trimmed reads were aligned to the Human genome (hg38) using STAR (53). Read counts for each gene were calculated using htseq-count (54) in conjunction with a gene annotation file for hg38 obtained from Ensembl (55). Low count transcripts (< 10 total across all samples) were filtered out. Genes on the Y chromosome were filtered out, as well, to remove variability introduced by sex differences in expression.

The data discussed in this publication have been deposited in NCBI’s Gene Expression Omnibus (56) and are accessible through GEO Series accession no. GSE218810 (https://www.ncbi.nlm.nih.gov/geo/query/acc.cgi?acc=GSE218810).

### Batch adjustment of RNA-Seq raw counts.

Raw counts generated by RNA-Seq with low count transcripts (< 10 total) filtered out were adjusted with the R package CombatSeq (57) to account for unwanted batch effects due to intraparticipant variation while preserving differences due to time of biopsy collection and intervention group. Upon identification of a factor involved in severity of response to NM exposure (Figure 3A, bottom), raw count adjustment with CombatSeq was repeated to account for intraparticipant variation with additional preservation of this effect.

### RNA-Seq exploratory data analysis and PCA.

Batch-adjusted counts (*n* = 110) were uploaded to the interactive companion tool pcaExplorer (58) in RStudio for PCA. Variance stabilized transformation was performed on the adjusted counts, and the top 5,000 variable genes were used to generate a PCA plot of the top 2 principal components. The 95% CI for labeled groups are shown as ellipses.

### RNA-Seq differential expression testing.

DESeq2 (59) was used to conduct a Wald test on adjusted counts using a design matrix that grouped samples by time of biopsy collection and intervention group. DEGs from comparisons between a group of interest and baseline were extracted from the results, and the log fold change (logFC) values were shrunk using the apeglm (60) method with a cutoff of 1 for visualization and gene ranking. Significance was determined by the “false sign or small” rate represented as an *S* < 0.005. Intragroups comparisons were extracted from the results of the Wald test, and DEGs with a log_2_FC cutoff of 1 and *P*_adj_ value (i.e., FDR) < 0.05 were obtained.

### DEG clustering by expression pattern and pathway enrichment.

The list of significant DEGs (log_2_FC ± 2 and *P*_adj_ < 0.05) identified between the Severe and Mild subgroups from the biopsy taken 3 days after intervention were filtered from variance-stabilized transformed counts. Clustering of DEGs according to expression profile across biopsies was executed with the degPatterns function from the R package DEGreport (61), which is visualized as the *Z* score of gene abundance across biopsies between groups. DEG lists from identified clusters were then subjected to KEGG pathway enrichment using the ClueGO plugin for Cytoscape (62).

### Cell type enrichment.

Normalized counts were extracted from the DESeq2 results object and subjected to the rawEnrichmentAnalysis function from the xCell (16) package in R for cell type enrichment analysis of curated cell type gene sets. Enrichment scores were submitted to the interactive xCell heatmap viewer (https://comphealth.ucsf.edu/app/xcellview/). Weak signatures were filtered out, and enrichment scores were normalized. Row and column dendrograms were generated using Euclidean distance and Ward.D linkage.

### Proximity extension assay.

Total protein was purified, quantified, and submitted to Olink for proximity extension assays utilizing the Olink “Inflammation panel” that can simultaneously probe 92 proteins in a single well. Normalized protein expression (NPX) output above the level of detection was exponentiated and adjusted according to input concentration, followed by log_2_ transformation of values back to NPX. Samples were run in 2 batches. The first batch consisted of all biopsies from baseline (*n* = 24) and from 72hrs P-E (*n* = 24) (control phase). The second batch consisted of all biopsies from baseline, 72hrs P-I (*n* = 24), and 6wks P-I (*n* = 23) (investigative phase).

### Identification of NM injury markers.

A 2-tailed Welch’s 2-sample *t* test at 95% CI was performed between baseline and 72hrs P-E for every protein. Multiple testing was corrected using the Benjamini-Hochberg method. The resulting list of significantly DEPs was used in post hoc analyses.

### Linear modeling of proteomic data.

NPX outputs from the 2 Olink Inflammation panel runs described above were subjected to linear mixed-effects modeling with olink_lmer_posthoc function. Adjusted *P* values were calculated using the Benjamini-Hochberg method from the p.adjust function in the stats R package. Investigative phase samples were grouped by biopsy and intervention group. Intraparticipant variation was treated as a random effect. The differential expression output was then used for downstream analyses. Upon identification of a factor that influences severity of response to NM, an additional grouping variable was used that accounts for this severity factor in all biopsies, with exception of those taken at baseline.

### Visualization of transcriptomic and proteomic data.

Volcano plots of DEPs and DEGs were generated in GraphPad Prism 9.4.1 using NPX differences and corresponding –log_10_ transformed *P*_adj_ values in comparisons of interest. Scatter plots of mean NPX values with SEM error bars were generated in GraphPad Prism 9.4.1. These values can be found in Supplemental Table 7. Significance between groups of interest were labeled according to the linear mixed-effects modeling results.

### Pathway enrichment analysis.

DEG and DEP lists were assessed for enrichment in annotated pathways from the KEGG database using the plugin ClueGO (62) in Cytoscape. Total number of DEPs or DEGs was taken into consideration when determining the stringency of input settings for the minimum number of markers, minimum percent markers per pathway term, and κ score cutoff (Supplemental Table 5). A κ score greater than the established cutoff will result in linkage between the 2 nodes, in which the label is determined by the most significantly enriched pathway of the group. Significant enrichment (*pV* < 0.05) was determined by a 2-sided hypergeometric test. The Bonferroni step-down method was used for multiple comparison correction. Significant enrichment of KEGG pathways was visualized as a pie chart in which functionally grouped networks of pathway enrichment are presented as a percentage of the total number of enriched pathways.

### Pathologic evaluation.

Biopsies were sectioned and stained with H&E, followed by imaging at 20***×*** magnification with a Zeiss microscope. Blinded histologic analysis was performed on all biopsy specimens by a single, board-certified dermatopathologist. Semiquantitative scores between 0 and 3 were assigned for each of the following metrics: degree of spongiosis, degree of interface change, and degree of inflammation. For all metrics, a score of 0 meant that the feature was absent. For degree of spongiosis, 1 indicates intercellular prominence without vesicle or bullae formation, 2 indicates vesicle formation, and 3 indicates bulla formation. For interface dermatitis, 1 indicates occasional basal vacuolization, 2 indicates necrotic/dyskeratotic cells, and 3 indicates full-thickness involvement. For inflammation, 1 indicates mild superficial perivascular infiltrate, 2 indicates moderate superficial perivascular infiltrate (20 or more cells), and 3 indicates dense perivascular infiltrate or moderate perivascular infiltrate with both superficial and deep involvement.

### Data and materials availability.

RNA and protein data are available in public databases (https://www.ncbi.nlm.nih.gov/geo/query/acc.cgi?acc=GSE218810). Protein data are available upon request.

### Statistics.

Two-way ANOVA followed by Tukey’s method to correct for multiple comparisons were performed in GraphPad Prism 9.4.1 to assess significant differences in serum VitD metabolite concentration (Supplemental Table 2), arm redness differences (Supplemental Table 8), and quantitative serum laboratory data obtained from blood draws (Supplemental Table 9).

### Study approval.

This early phase I clinical trial was conducted in accordance with the Declaration of Helsinki guidelines, registered on clinicaltrials.gov (NCT02968446), and approved by the IRB of Case Western Reserve University and Case Comprehensive Cancer Center (CASE 3416). All participants provided written consent prior to participation. This study was funded by the NIH and National Institute of Arthritis and Musculoskeletal and Skin Diseases (NIAMS; grants U01AR064144-01, U01AR071168, and U01AR075049).

## Author contributions

This work was conceptualized by KQL and KDC. Methodology was determined by KQL, KDC, STE, MKE, LCT, and JEG. JMT, STE, UVO, DB, MMD, CVN, LFC, TSM, RMR, and KSH contributed to study investigation. Visualization of results was performed by STE and MKE. Funding was acquired by KQL. Project was supervised by KQL. The original manuscript draft was written by MKE, STE, and KQL. The manuscript was reviewed and edited by MKE, STE, RMR, UVO, MMD, DB, KQL, KDC, JEG, and LCT. MKE and STE are co–first authors and contributed equally. The ordering of names was decided alphabetically.

## Supplementary Material

Supplemental data

ICMJE disclosure forms

Supplemental table 3

Supplemental table 4

Supplemental table 5

Supplemental table 6

Supplemental table 7

Supplemental table 8

Supplemental table 9

## Version 1 01/24/2023

Electronic publication

## References

[1] Vonderheid EC (1997). Topical chemotherapy and immunotherapy of mycosis fungoides: intermediate-term results. Arch Dermatol.

[2] Estève E (1999). A prospective study of cutaneous intolerance to topical mechlorethamine therapy in patients with cutaneous T-cell lymphomas. French Study Group of cutaneous lymphomas. Arch Dermatol.

[3] De Quatrebarbes J (2055). Treatment of early-stage mycosis fungoides with twice-weekly applications of mechlorethamine and topical corticosteroids: a prospective study. Arch Dermatol.

[4] Kim YH (1996). Clinical stage IA (limited patch and plaque) mycosis fungoides. A long-term outcome analysis. Arch Dermatol.

[5] Aranow C (2011). Vitamin D and the immune system. J Investig Med.

[6] Di Rosa M (2012). Immuno-modulatory effects of vitamin D3 in human monocyte and macrophages. Cell Immunol.

[7] Das LM (2019). Vitamin D improves sunburns by increasing autophagy in M2 macrophages. Autophagy.

[8] Au L (2015). Suppression of hyperactive immune responses protects against nitrogen mustard injury. J Invest Dermatol.

[9] Highton A (1995). Calcipotriene ointment 0.005% for psoriasis: a safety and efficacy study. Calcipotriene study group. J Am Acad Dermatol.

[10] Scott JF (2017). Oral vitamin D rapidly attenuates inflammation from sunburn: an interventional study. J Invest Dermatol.

[11] Biyashev D (2020). A novel treatment for skin repair using a combination of spironolactone and vitamin D3. Ann N Y Acad Sci.

[12] Wennberg E (2021). Topical treatments for early-stage mycosis fungoides using grading recommendations assessment, development and evaluation (grade) criteria: a systematic review. JAAD Int.

[13] Kim YH (2003). Topical nitrogen mustard in the management of mycosis fungoides: update of the Stanford experience. Arch Dermatol.

[14] Lessin SR (2013). Topical chemotherapy in cutaneous T-cell lymphoma: positive results of a randomized, controlled, multicenter trial testing the efficacy and safety of a novel mechlorethamine, 0.02%, gel in mycosis fungoides. JAMA Dermatol.

[15] Pludowski P (2018). Vitamin D supplementation guidelines. J Steroid Biochem Mol Biol.

[16] Aran D (2017). xCell: digitally portraying the tissue cellular heterogeneity landscape. Genome Biol.

[17] Cannons JL (2001). 4-1BB ligand induces cell division, sustains survival, and enhances effector function of CD4 and CD8 T cells with similar efficacy. J Immunol.

[18] Watts TH (2005). TNF/TNFR family members in costimulation of T cell responses. Annu Rev Immunol.

[19] Takahashi C (1999). Cutting edge: 4-1BB is a bona fide CD8 T cell survival signal. J Immunol.

[20] Halstead ES (2002). In vivo stimulation of CD137 broadens primary antiviral CD8+ T cell responses. Nat Immunol.

[21] Tsou C-L (2007). Critical roles for CCR2 and MCP-3 in monocyte mobilization from bone marrow and recruitment to inflammatory sites. J Clin Invest.

[22] Flier J (2001). Differential expression of CXCR3 targeting chemokines CXCL10, CXCL9, and CXCL11 in different types of skin inflammation. J Pathol.

[23] Tensen CP (1999). Human IP-9: a keratinocyte-derived high affinity CXC-chemokine ligand for the IP-10/Mig receptor (CXCR3). J Invest Dermatol.

[24] Rogers JV (2005). Microarray analysis of gene expression in murine skin exposed to sulfur mustard. J Biochem Mol Toxicol.

[25] Venosa A (2016). Characterization of distinct macrophage subpopulations during nitrogen mustard-induced lung injury and fibrosis. Am J Respir Cell Mol Biol.

[26] Venosa A (2015). Protective role of spleen-derived macrophages in lung inflammation, injury, and fibrosis induced by nitrogen mustard. Am J Physiol Lung Cell Mol Physiol.

[27] Bhattarai HK (2020). Vitamin D, calcium, parathyroid hormone, and sex steroids in bone health and effects of aging. J Osteoporos.

[28] Gerecke DR (2009). Differential gene expression profiling of mouse skin after sulfur mustard exposure: extended time response and inhibitor effect. Toxicol Appl Pharmacol.

[29] Chang Y-C (2018). Expression of cytokines and chemokines in mouse skin treated with sulfur mustard. Toxicol Appl Pharmacol.

[30] Wolf M (2016). Characterization of sulfur mustard resistant keratinocyte cell line HaCaT/SM. Toxicol Lett.

[31] Price JA (2009). Transcriptional changes in porcine skin at 7 days following sulfur mustard and thermal burn injury. Cutan Ocul Toxicol.

[32] Achanta S (2018). TRPA1 and CGRP antagonists counteract vesicant-induced skin injury and inflammation. Toxicol Lett.

[33] Egeberg A (2020). Systematic review on rapidity of onset of action for interleukin-17 and interleukin-23 inhibitors for psoriasis. J Eur Acad Dermatol Venereol.

[34] Milatovic S (2003). Impaired healing of nitrogen mustard wounds in CXCR2 null mice. Wound Repair Regen.

[35] Carr MW (1994). Monocyte chemoattractant protein 1 acts as a T-lymphocyte chemoattractant. Proc Natl Acad Sci U S A.

[36] Nakamura N (1995). Keratinocyte-derived monocyte chemoattractant protein 1 (MCP-1): analysis in a transgenic model demonstrates MCP-1 can recruit dendritic and Langerhans cells to skin. J Invest Dermatol.

[37] Harper EG (2009). Th17 cytokines stimulate CCL20 expression in keratinocytes in vitro and in vivo: implications for psoriasis pathogenesis. J Invest Dermatol.

[38] Hoffmann E (2002). Multiple control of interleukin-8 gene expression. J Leukoc Biol.

[39] Teijeira A (2021). Differential interleukin-8 thresholds for chemotaxis and netosis in human neutrophils. Eur J Immunol.

[40] Mishra NC (2012). Inhalation of sulfur mustard causes long-term T cell-dependent inflammation: possible role of Th17 cells in chronic lung pathology. Int Immunopharmacol.

[41] Hijnen D (2013). CD8(+) T cells in the lesional skin of atopic dermatitis and psoriasis patients are an important source of IFN-γ, IL-13, IL-17, and IL-22. J Invest Dermatol.

[42] Tzartos JS (2008). Interleukin-17 production in central nervous system-infiltrating T cells and glial cells is associated with active disease in multiple sclerosis. Am J Pathol.

[43] Ortega C (2009). IL-17-producing CD8+ T lymphocytes from psoriasis skin plaques are cytotoxic effector cells that secrete Th17-related cytokines. J Leukoc Biol.

[44] Mashiko S (2015). Human mast cells are major IL-22 producers in patients with psoriasis and atopic dermatitis. J Allergy Clin Immunol.

[45] Brembilla N (2017). IL-17A localizes in the exocytic compartment of mast cells in psoriatic skin. Br J Dermatol.

[46] Barin JG (2012). Macrophages participate in IL-17-mediated inflammation. Eur J Immunol.

[47] Chen J (2013). IL-17A induces pro-inflammatory cytokines production in macrophages via MAPKinases, NF-κB and AP-1. Cell Physiol Biochem.

[48] Adams JS (1983). Metabolism of 25-hydroxyvitamin D3 by cultured pulmonary alveolar macrophages in sarcoidosis. J Clin Invest.

[49] Adams JS (1985). Isolation and structural identification of 1,25-dihydroxyvitamin D3 produced by cultured alveolar macrophages in sarcoidosis. J Clin Endocrinol Metab.

[50] Amrein K (2014). Effect of high-dose vitamin D3 on hospital length of stay in critically ill patients with vitamin D deficiency: the VITdAL-ICU randomized clinical trial. JAMA.

[51] Von Restorff C (2009). High-dose oral vitamin D3 supplementation in rheumatology patients with severe vitamin D3 deficiency. Bone.

[52] Martin M (2011). Cutadapt removes adapter sequences from high-throughput sequencing reads. EMBnet J.

[53] Dobin A (2013). STAR: ultrafast universal RNA-seq aligner. Bioinformatics.

[54] Anders S (2015). HTSeq--a Python framework to work with high-throughput sequencing data. Bioinformatics.

[55] Howe KL (2020). Ensembl 2021. Nucleic Acids Res.

[56] Edgar R (2002). Gene expression omnibus: NCBI gene expression and hybridization array data repository. Nucleic Acids Res.

[57] Zhang Y (2020). ComBat-seq: batch effect adjustment for RNA-seq count data. NAR Genom Bioinform.

[58] Marini F, Binder H (2019). pcaExplorer: an R/Bioconductor package for interacting with RNA-seq principal components. BMC Bioinformatics.

[59] Love MI (2014). Moderated estimation of fold change and dispersion for RNA-seq data with DESeq2. Genome Biol.

[60] Zhu A (2018). Heavy-tailed prior distributions for sequence count data: removing the noise and preserving large differences. Bioinformatics.

[61] http://lpantano.github.io/DEGreport/.

[62] Bindea G (2009). ClueGO: a Cytoscape plug-in to decipher functionally grouped gene ontology and pathway annotation networks. Bioinformatics.

[63] Apisarnthanarax N (2012). Phase I clinical trial of O6-benzylguanine and topical carmustine in the treatment of cutaneous T-cell lymphoma, mycosis fungoides type. Arch Dermatol.

[64] Osuka A (2014). Immune response to traumatic injury: harmony and discordance of immune system homeostasis. Acute Med Surg.

[65] Zelenay S, e Sousa CR (2013). Adaptive immunity after cell death. Trends Immunol.

[66] Sørensen OE, Borregaard N (2016). Neutrophil extracellular traps—the dark side of neutrophils. J Clin Invest.

[67] Remick DG (2005). Interleukin-8. Crit Care Med.

[68] Colonna M (2004). Plasmacytoid dendritic cells in immunity. Nat Immunol.

[69] Tillack K (2012). T lymphocyte priming by neutrophil extracellular traps links innate and adaptive immune responses. J Immunol.

[70] Qiu S-L (2017). Neutrophil extracellular traps induced by cigarette smoke activate plasmacytoid dendritic cells. Thorax.

[71] Kaplan MJ, Radic M (2012). Neutrophil extracellular traps: double-edged swords of innate immunity. J Immunol.

[72] Soni C (2020). Plasmacytoid dendritic cells and type I interferon promote extrafollicular B cell responses to extracellular self-DNA. Immunity.

