# Supplementary material for: Vitamin D3 and deconvoluting a rash
Source: JCI Insight. 2023 Jan 24;8(2):e163789. doi: 10.1172/jci.insight.163789 (PMC9977299; doi:10.1172/jci.insight.163789)
Supplement: Supplemental data [file jciinsight-8-163789-s199.pdf]

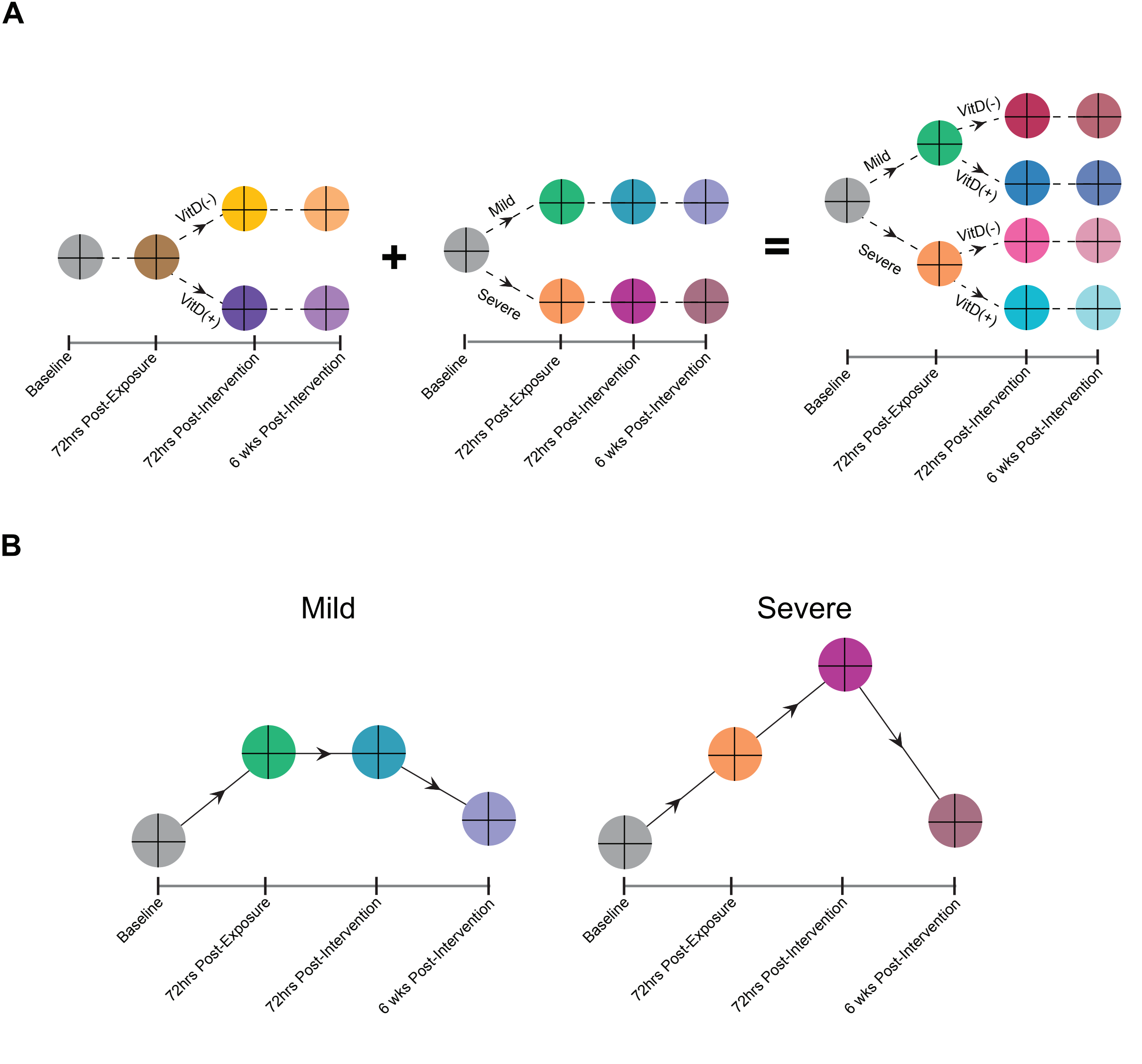

Supplemental Figure 1. Schematic of sample grouping for DE testing in RNA-seq and proteomics. (A) Intersected circles represent time points at which biopsies were taken and the intervention group (VitD(-), VitD(+)) as well as response type (Mild, Severe) are taken into account. (B) Hypothesized expression profile of markers induced by NM-injury (Mild) as well as those that had an exaggerated response upon iterative NM exposure (Severe).

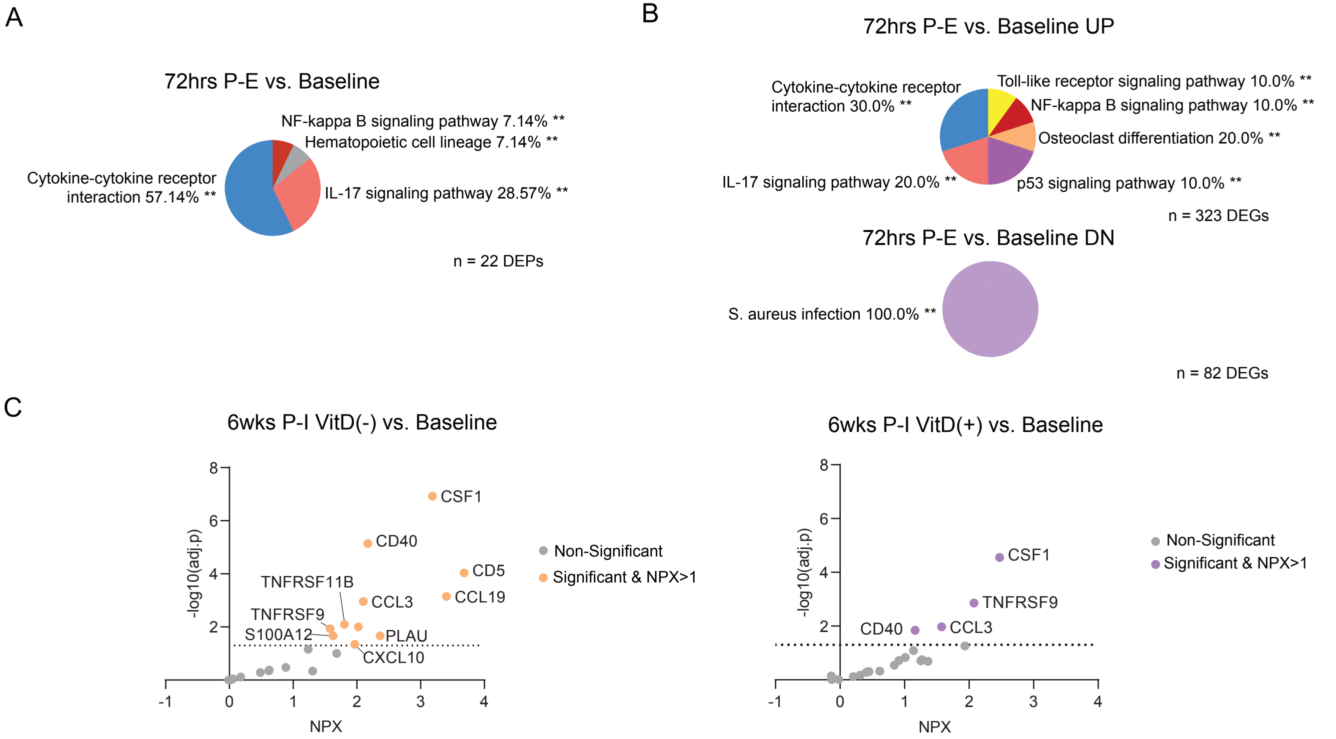

Supplemental Figure 2 Exposure to nitrogen mustard induces IL-17 signaling genes and proteins. (A) Pie chart shows KEGG pathway enrichment using 22 differentially expressed proteins (adj. p<0.05) in samples from 72hrs post-exposure and baseline. (B) Pie charts of KEGG pathway enrichment using 323 upregulated (Log2FC>1, s-value<0.005) DEGs and 82 downregulated DEGs. (C) Volcano plots of NM-injury marker NPX differences in 6wks P-I relative to baseline for both VitD(-) and VitD(+). (A,B) **pV<0.01.

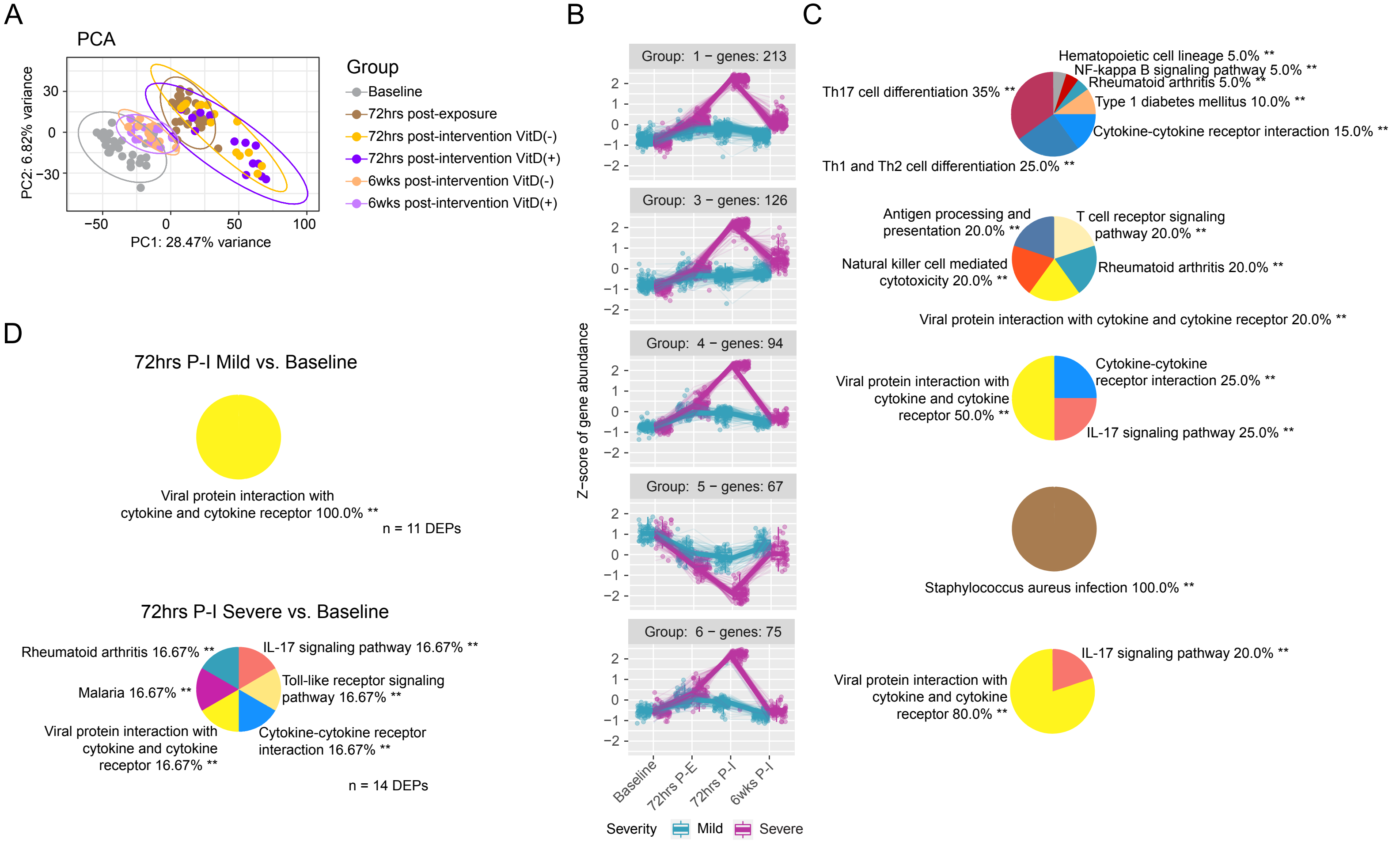

Supplemental Figure 3. Divergent response to NM is intervention-independent and involves IL-17 signaling and Th cell differentiation. A. PCA of RNA-seq grouped by biopsy and intervention group. Ellipses denote 95% confidence intervals. (B) Plots of z-score transformed normalized RNA-seq counts for DEGs (adj. p<0.05, Log2FC cutoff = 2) found in 72hrs P-I between Mild (n=14) and Severe (n=14) subgroups. (C) Pie charts show the percent composition of DEG clusters found in (B). (D) Pie charts show the percent composition of significantly enriched KEGG pathways using NM injury DEP lists as input from comparisons between 72hrs P-I Severe and Mild relative to baseline. Significant enrichment was determined by a two-sided hypergeometric test and p-values were corrected using Bonferroni step down. (C,D) **pV<0.01

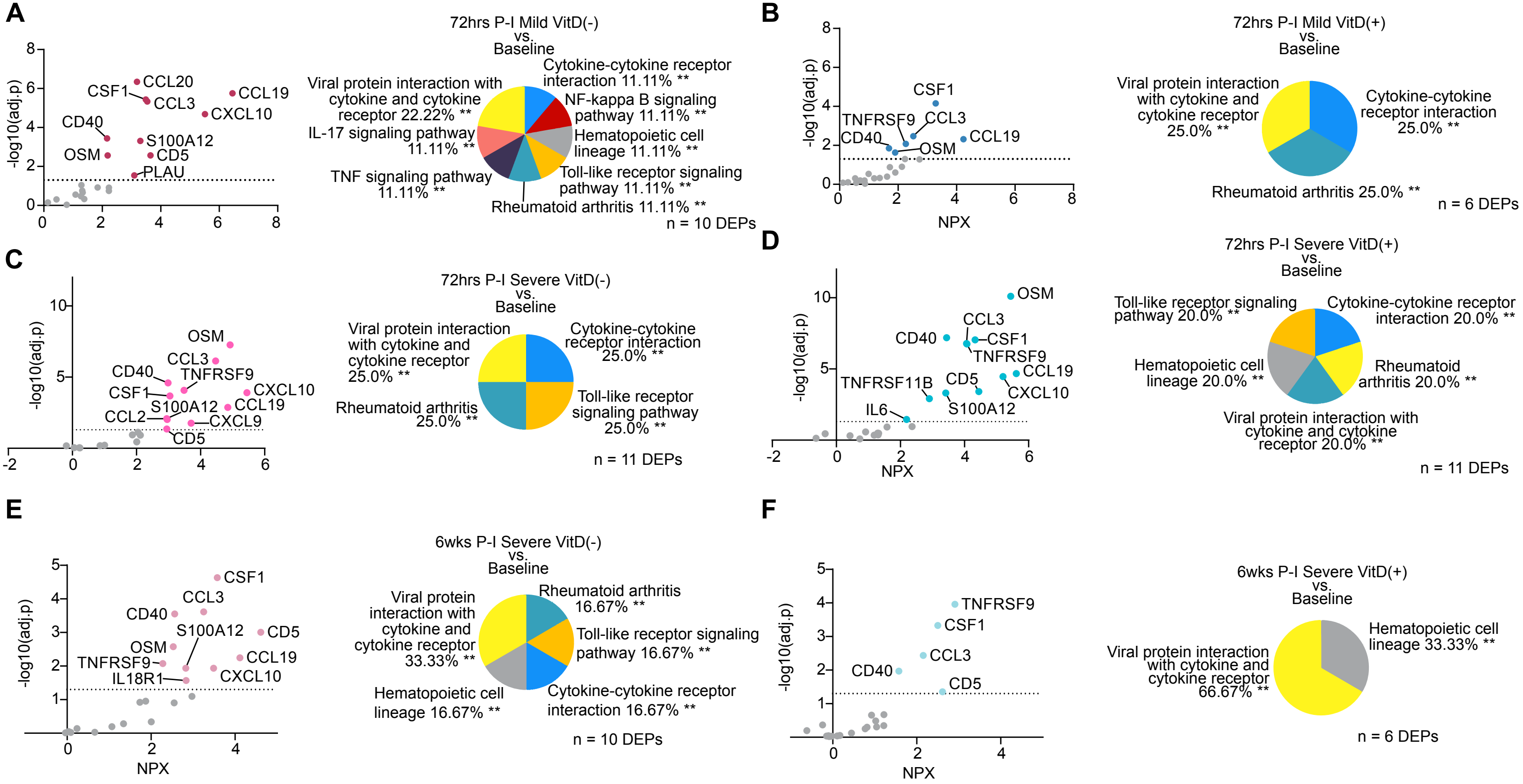

Supplemental Figure 4. VitD intervention suppresses acute IL-17 signaling in Mild responders and resolves longitudinal NM-injury in Severe responders. (A) Volcano plot of NM-injury marker NPX differences between 72hrs P-I Mild VitD(-) (n=7) and Baseline (n=24). Pie chart of significantly enriched KEGG pathways using significant NM-injury markers. (B) Volcano plot of NM-injury marker NPX differences between 72hrs P-I Mild VitD(+) (n=5) and Baseline (n=24). Pie chart of significantly enriched KEGG pathways using significant NM-injury markers. (C) Volcano plot of NM-injury marker NPX differences between 72hrs P-I Severe VitD(-) (n=5) and Baseline (n=24). Pie chart of significantly enriched KEGG pathways using significant NM-injury markers. (D) Volcano plot of NM-injury marker NPX differences between 72hrs P-I Severe VitD(+) (n=7) and Baseline (n=24). Pie chart of significantly enriched KEGG pathways using significant NM-injury markers. (E) Volcano plot of NM-injury marker NPX differences between 6wks P-I Severe VitD(-) (n=5) and Baseline (n=24). Pie chart of significantly enriched KEGG pathways using significant NM-injury markers. (F) Volcano plot of NM-injury marker NPX differences between 6wks P-I Severe VitD(+) (n=7) and Baseline (n=24). Pie chart of significantly enriched KEGG pathways using significant NM-injury markers.

Supplemental Table 1. Subject Demographics

Subject ID	Intervention Group	Gender	Race	Fitzpatrick Skin Type	Ethnicity	Age	BMI
AN007	VitD(-)	Female	White	?	Non-hispanic/Latino	36	38.77
AN011	VitD(+)	Female	Black/African American	3	Non-hispanic/Latino	37	25.36
AN014	VitD(+)	Male	White	2	Non-hispanic/Latino	39	36.05
AN017	VitD(-)	Female	White	2	Non-hispanic/Latino	41	31.60
AN020	VitD(-)	Female	White	2	Non-hispanic/Latino	43	30.66
AN028	VitD(+)	Male	White	3	Non-hispanic/Latino	30	25.38
AN029	VitD(+)	Female	White	1	Non-hispanic/Latino	23	20.04
CN024	VitD(+)	Male	White	3	Non-hispanic/Latino	20	22.72
EN005	VitD(+)	Female	White	?	Non-hispanic/Latino	23	18.54
EN012	VitD(-)	Female	White	2	Hispanic/Latino	22	24.75
EN016	VitD(+)	Female	Black/African American	4	Non-hispanic/Latino	47	31.79
EN030	VitD(-)	Female	White	4	Non-hispanic/Latino	24	18.93
GN003	VitD(-)	Female	Black/African American	?	Unknown	27	25.11
HN031	VitD(+)	Male	Asian	3	Non-hispanic/Latino	25	22.51
JN015	VitD(-)	Female	White	2	Non-hispanic/Latino	61	23.23
JN032	VitD(-)	Male	White	2	Non-hispanic/Latino	49	32.28
JN033	VitD(+)	Male	Asian	4	Non-hispanic/Latino	25	22.15
KN013	VitD(-)	Female	White	2	Non-hispanic/Latino	23	24.02
KN022	VitD(+)	Female	White	2	Non-hispanic/Latino	25	20.23
KN025	VitD(+)	Female	White	3	Non-hispanic/Latino	39	25.51
MN004	VitD(+)	Male	White	?	Unknown	64	24.27
MN021	VitD(-)	Female	White	2	Non-hispanic/Latino	27	24.01
NN023	VitD(-)	Male	White	3	Non-hispanic/Latino	27	25.99
RN034	VitD(-)	Female	White	2	Non-hispanic/Latino	25	24.36
SN009	VitD(+)	Female	White	2	Non-hispanic/Latino	56	38.04
SN019	VitD(+)	Female	White	2	Non-hispanic/Latino	26	23.43
SN026	VitD(-)	Female	White	2	Non-hispanic/Latino	35	24.10
TN027	VitD(-)	Female	Black/African American	5&6	Non-hispanic/Latino	48	25.22

Supplemental Table 2. Serum Vitamin D metabolite levels

	25(OH)2D				
**VitD(-)**	**Baseline**	**24hrs P-I**	**48hrs P-I**	**72hrs P-I**	**168hrs P-I**
AN007	27	27.4	26.5	26.2	23.6
AN017	33.5	31.7	30.2	33.2	29.3
EN012	34.3	30.2	31.1	31.3	32.5
JN032	28.7	29.1	27.8	28.5	26.7
MN021	29.9	27.9	28.1	26.5	24.9
NN023	16.8	20.4	17.6	17.5	19.3
RN034	35.6	33.7	35.4	33.5	29
TN027	18.6	21.5	23.1	23.3	27.1
AN020	23.8	21.7	22.5	22.8	24.2
EN030	40.9	38.8	40.1	41.7	37.5
GN003	13.6	13	12.9	13	12.6
JN015	36.3	38.7	35.8	37.5	35
KN013	25	26.1	24.9	27.5	29.1
SN026	31.1	30	31.8	35.1	28
**Average**	28.2214286	27.8714286	27.7	28.4	27.0571429
**SD**	7.96542322	7.102592	7.29583654	7.76907083	6.26169236
**SEM**	2.1288489	1.89824756	1.94989433	2.07637152	1.67350768

**VitD(+)**	**Baseline**	**24hrs P-I**	**48hrs P-I**	**72hrs P-I**	**168hrs P-I**
AN028	21	34.6	34.8	42	43.5
CN024	19	36.8	47.9	53	58.9
EN016	21.6	40.2	46.9	50	47.3
HN031	13.5	43.6	54.3	60.6	58.7
MN004	31.2	39.3	42.3	45.3	45.5
SN009	21.1	29.2	32.8	31.3	34.8
AN011	29.7	42.8	45.1	52.4	50.2
AN014	36.3	44.8	50.3	50.8	54.3
AN029	13.4	31	39.6	43.5	43.1
EN005	33.1	64.2	66.7	71.7	77.1
JN033	17.7	35.8	44	42.1	47.5
KN022	24.3	53.9	66.9	70.4	70.2
KN025	25.3	39.8	44.8	45.7	52.3
SN019	17.9	34.9	38	41.7	42.7
**Average**	23.2214286	40.7785714	46.7428571	50.0357143	51.8642857
**SD**	7.12203283	9.17967475	10.2805054	11.2407642	11.3696362
**SEM**	1.90344334	2.45337127	2.74758064	3.00422061	3.03866308

	1,25(OH)2D				
**VitD(-)**	**Baseline**	**24hrs P-I**	**48hrs P-I**	**72hrs P-I**	**168hrs P-I**
AN007	57.3	29.7	34.8	48.1	43.2
AN017	44	37.3	32.2	35	40.2
EN012	64.8	63.7	50.6	53.8	56.5
JN032	35.8	39.4	40.4	44.6	41.6
MN021	65.7	56.2	52.6	36.5	44.7
NN023	42.6	48	43.7	39.6	53.1
RN034	48.7	50.4	45.8	53.9	37
TN027	37.7	35.8	42.3	50.7	26.2
AN020	38.2	23.1	25	40.4	42.7
EN030	74.2	68.5	69.1	55.5	57.1
GN003	51.5	35.4	38.3	43.4	47.7
JN015	57.1	62.4	64.7	61.9	46.7
KN013	42.9	63.5	64.5	49.1	34.5
SN026	57.9	58.2	45.6	49.1	49.6
**Average**	51.3142857	47.9714286	46.4	47.2571429	44.3428571
**SD**	11.8658435	14.5240611	12.8743633	7.75685447	8.50553795
**SEM**	3.17128007	3.88171861	3.44081833	2.07310656	2.27320064

**VitD(+)**	**Baseline**	**24hrs P-I**	**48hrs P-I**	**72hrs P-I**	**168hrs P-I**
AN028	40.8	41.2	48.5	53.2	47.2
CN024	44.9	74.4	70.5	64.9	61.4
EN016	54.6	64.9	53	54.8	87.7
HN031	46.9	65.4	78.5	60	59
MN004	57.9	65.9	72.2	70.2	63.8
SN009	50.6	86	101	102	73
AN011	60	94.8	87	80.9	73
AN014	46.5	70.5	54.4	45	62.2
AN029	64.2	103	97.6	94.8	71.3
EN005	90.4	90.6	74.5	78.5	87
JN033	41.8	82.6	80.1	72.2	65.1
KN022	86.8	112	92.8	77.2	80.3
KN025	54.8	84.3	72.8	67.8	97
SN019	59.3	81.5	64.9	68.2	83.1
**Average**	57.1071429	79.7928571	74.8428571	70.6928571	72.2214286
**SD**	15.1253462	18.0049764	16.2464535	15.5723356	13.5807657
**SEM**	4.04241882	4.81203236	4.34204735	4.16188176	3.62961232

	24,25(OH)2D				
**VitD(-)**	**Baseline**	**24hrs P-I**	**48hrs P-I**	**72hrs P-I**	**168hrs P-I**
AN007	1.4	1.5	1.5	1.4	1.3
AN017	2.1	1.8	2.2	2.2	2.1
EN012	2.2	2.2	2	2.1	2.3
JN032	2.3	2.4	2.4	2.3	2.2
MN021	2	2	2.1	1.9	2.1
NN023	0.5	0.6	0.8	0.6	0.7
RN034	0.9	1	0.9	1	0.9
TN027	1	1.4	1.4	1.4	1.7
AN020	1.7	1.8	1.6	1.6	1.6
EN030	4.3	4.3	4.1	4.3	4.2
GN003	0.8	0.7	0.8	0.7	2.3
JN015	3	3.5	3.3	3.3	3.3
KN013	1.6	1.5	1.5	1.6	1.6
SN026	3.2	3.1	3.4	3.4	3.1
**Average**	1.92857143	1.98571429	2	1.98571429	2.1
**SD**	1.04912313	1.05746951	1.01451011	1.06616288	0.94624765
**SEM**	0.28038995	0.28262061	0.27113923	0.28494402	0.25289532

**VitD(+)**	**Baseline**	**24hrs P-I**	**48hrs P-I**	**72hrs P-I**	**168hrs P-I**
AN028	1	1.4	1.9	2.2	2.8
CN024	1.1	1.5	2.5	3.4	4.7
EN016	1.6	2.5	3.3	4.1	7.9
HN031	0.7	1.7	3.1	3.5	4.4
MN004	3.6	3.6	5.3	4.1	3.9
SN009	1.5	1.7	2	2.6	2.8
AN011	3	2.4	3.6	4.5	5.1
AN014	3.7	3.7	4.6	3.8	4.7
AN029	0.3	0.9	1.7	2.2	2.8
EN005	3.3	6	5	7.1	12.2
JN033	0.9	1.4	2.2	2.5	3.6
KN022	1.4	2.4	4	4.9	6.2
KN025	1.4	1.8	4.1	2.9	3.5
SN019	1.2	1.4	2	2.5	2.9
**Average**	1.76428571	2.31428571	3.23571429	3.59285714	4.82142857
**SD**	1.13449897	1.34155888	1.22575386	1.33501039	2.57418503
**SEM**	0.3032076	0.35854669	0.3275965	0.35679654	0.68797989

Supplemental Tables 3-9 are large excel files that can be found as uploaded "Supplemental Files"
